# Microcircuits for spatial coding in the medial entorhinal cortex

**DOI:** 10.1152/physrev.00042.2020

**Published:** 2021-07-13

**Authors:** John J. Tukker, Prateep Beed, Michael Brecht, Richard Kempter, Edvard I. Moser, Dietmar Schmitz

**Affiliations:** ^1^German Center for Neurodegenerative Diseases (DZNE) Berlin, Berlin, Germany; ^2^Neuroscience Research Center, Charité–Universitätsmedizin Berlin, corporate member of Freie Universität Berlin and Humbold-Universität zu Berlin, Berlin, Germany; ^3^Berlin Institute of Health at Charité–Universitätsmedizin Berlin, Berlin, Germany; ^4^Institute for Theoretical Biology, Humboldt-Universität zu Berlin, Berlin, Germany; ^5^Bernstein Center for Computational Neuroscience, Humboldt-Universität zu Berlin, Berlin, Germany; ^6^Neurocure Cluster of Excellence, Charité–Universitätsmedizin Berlin, corporate member of Freie Universität Berlin and Humboldt-Universität zu Berlin, Berlin, Germany; ^7^Einstein Center for Neurosciences Berlin, Charité–Universitätsmedizin Berlin, corporate member of Freie Universität Berlin and Humboldt-Universität zu Berlin, Berlin, Germany; ^8^Kavli Institute for Systems Neuroscience and Centre for Neural Computation, Norwegian University of Science and Technology, Trondheim, Norway

**Keywords:** connectivity, entorhinal cortex, grid cells, microcircuits, navigation

## Abstract

The hippocampal formation is critically involved in learning and memory and contains a large proportion of neurons encoding aspects of the organism’s spatial surroundings. In the medial entorhinal cortex (MEC), this includes grid cells with their distinctive hexagonal firing fields as well as a host of other functionally defined cell types including head direction cells, speed cells, border cells, and object-vector cells. Such spatial coding emerges from the processing of external inputs by local microcircuits. However, it remains unclear exactly how local microcircuits and their dynamics within the MEC contribute to spatial discharge patterns. In this review we focus on recent investigations of intrinsic MEC connectivity, which have started to describe and quantify both excitatory and inhibitory wiring in the superficial layers of the MEC. Although the picture is far from complete, it appears that these layers contain robust recurrent connectivity that could sustain the attractor dynamics posited to underlie grid pattern formation. These findings pave the way to a deeper understanding of the mechanisms underlying spatial navigation and memory.

## Graphical abstract

**Figure:**
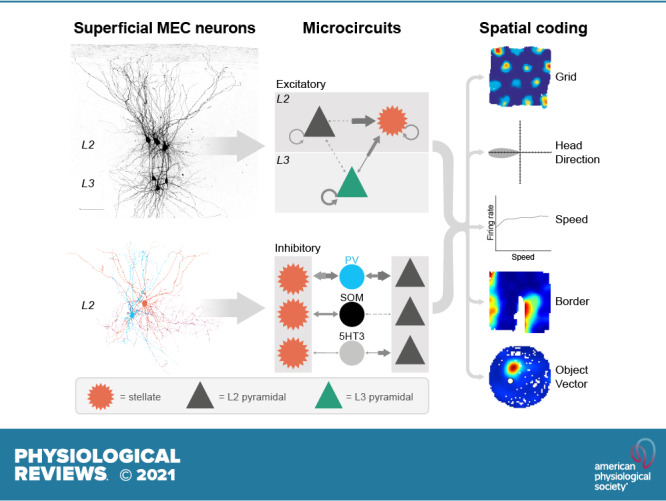

CLINICAL HIGHLIGHTS

- In humans, spatial coding is present in the entorhinal cortex with gridlike representations of place as well as direction, speed, and boundary or vectorlike representations (1–9).
- MEC dysfunction underlies deficits in spatial navigation and semantic, episodic, and working memory, potentially causing symptoms in a wide range of disorders (10–22).
- In Alzheimer’s disease (AD), both interneurons and principal cells in superficial MEC are affected at very early stages (11, 12, 23–27).
- Even before the onset of proper AD, path integration is affected in patients with mild cognitive impairment and can be used as a biomarker to predict AD (28). Path integration deficits may also reflect more general memory deficits and likely contribute to the difficulties AD patients experience in navigation (29–32).
- Path integration deficits correlate with grid cell-like activity in the entorhinal cortex in aged humans (33), and distance estimation errors in human path integration can be predicted by a grid cell model (34).
- In temporal lobe epilepsy, epileptiform activity may originate in superficial MEC, particularly in the highly excitable L3 pyramidal neurons. These cells are selectively degenerated both in human patients postmortem and in rat models of epilepsy (15, 35, 36). Deficits in MEC may also underlie the cognitive deficits often associated with epilepsy.
- Deep brain stimulation of the MEC, but not the hippocampus, can directly improve spatial learning (37–39).
- We envision more refined therapies in the future, which build on our exquisite knowledge of the MEC, specifically targeting memory deficits based on our understanding of the underlying connectivity.

## 1. INTRODUCTION

**FIGURE 1.**
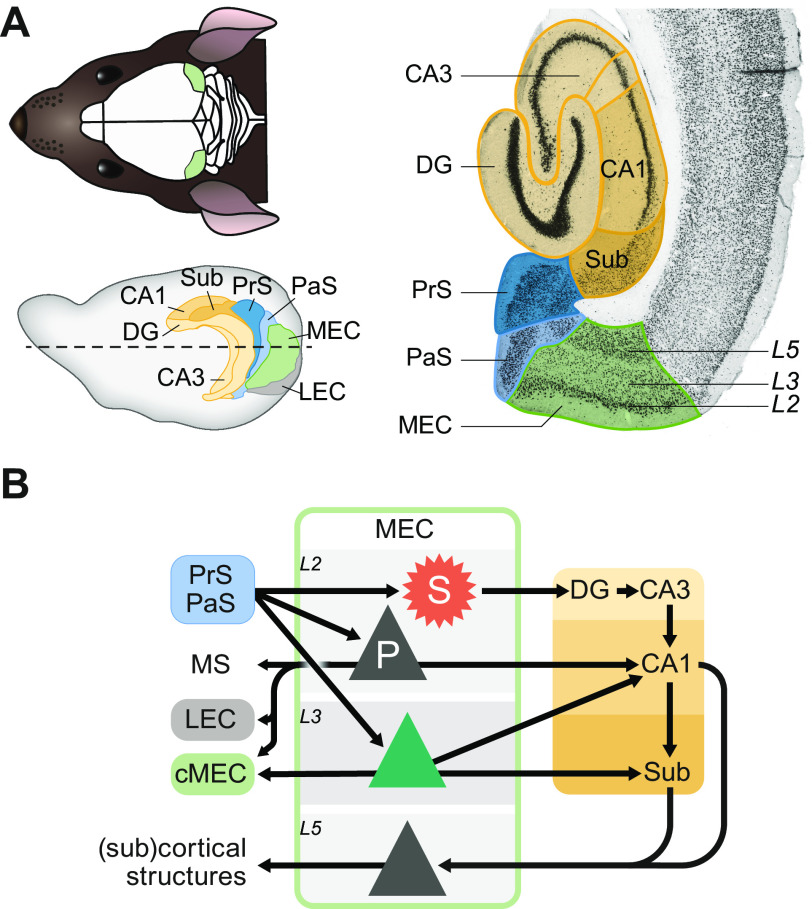
Medial entorhinal cortex (MEC) anatomy and extrinsic connections. *A*: mouse head (*top left*) showing the location of the MEC (green) at the posterior edge of the cortex and a sagittal view of the rat brain (*bottom left*) showing the MEC and main connected structures including the lateral entorhinal cortex (LEC, gray), hippocampus (yellow), and presubiculum (PrS) and parasubiculum (PaS) (both blue). Dashed line depicts the horizontal plane for the brain section on *right*, which also shows layer (L)2, L3, and L5 in the MEC. DG, dentate gyrus; Sub, subiculum. *B*: main inputs to and outputs from principal cells in the MEC, including stellate (S, red) and pyramidal (P, gray) cells in L2 and pyramidal cells in L3 (green) and L5 (gray). Arrows depict main known excitatory connections. Note the strong output from stellate cells in L2 and pyramidal cells in L3 to the hippocampus (yellow), which provides the main input to L5 pyramidal cells. Inputs to the superficial MEC are mostly from the PrS and PaS. cMEC, contralateral MEC; MS, medial septum; further colors and abbreviations as in *A*. Data summarized are from Refs. 49, 50, 58–60, 65, 85–87). *Bottom left* and *right* images in *A* adapted from Moser et al. (53) with permission from *Nature Reviews Neuroscience*.

The medial entorhinal cortex (MEC) is extensively connected with the hippocampus and many other cortical and subcortical areas (40–43) (FIGURE 1). A series of recent in vitro physiological studies have started to elucidate the short-range connectivity within the MEC, both within and across cortical layers (44–52). Here, we attempt to bridge the gap between these in vitro studies, which highlight the local connections between particular anatomically defined cell types, and in vivo studies from behaving rodents, which have described spatial coding in the same MEC circuit (53–55).

We start by describing the various elements that make up the MEC microcircuitry at two levels: functionally defined cell types and anatomically defined cell types. Functionally defined cell types encoding spatial information include grid, head direction (HD), speed, border, and object-vector (OV) cells, as well as various types of conjunctive cells, i.e., cells with mixed firing correlates. We include some key experiments that show correlations and dissociations between particular subsets of these cell types, which suggest particular constraints on local connectivity. Because synaptic connectivity studies are still based on anatomically defined cell types, we include a brief review of the main anatomically defined cell types in the superficial MEC in terms of morphology, hodology, and intrinsic properties. Although the superficial and deep layers of MEC are interconnected, we focus on the superficial layers because in the deep layers there is thus far very little data on microcircuits. Furthermore, the superficial MEC provides the major input to the hippocampus (FIGURE 1*B*), and one motivation for studying superficial MEC microcircuits has been to explain spatial coding in the hippocampus, where cells firing at specific places in the environment (“place cells”) were first described 50 years ago (56, 57).

Of course, knowing which cell types exist in the circuit is an important first step, but to understand how function is generated it is also crucial to know which types of neurons are connected (and which ones are not), how strong and how common such connections are, and how these connections can change over time to enable learning. Although we are still in the early stages of addressing these questions, recent advances have been made in describing the connections of neurons in the superficial MEC. These connections include two broad classes: synaptic connections between various anatomically or genetically defined cell types, as revealed by studies using brain slices, anatomical tracing, or ultrastructural reconstruction (13, 44–48, 50–52, 58–60), and functional connections between genetically and functionally defined cell types, as deduced from in vivo extracellular recordings in combination with chemo- and optogenetics (61–65) or calcium imaging (66, 67).

We discuss the extent to which the structure of the MEC microcircuit is compatible with the existence of continuous-attractor network (CAN) dynamics in the superficial MEC, which has been proposed to underlie gridlike and head directional firing patterns (see appendix). CAN theories propose that cells with similar tuning properties are more interconnected than dissimilar cells, such that *1*) cells with similar tuning can sustain activity in the absence of external inputs and *2*) these “bumps” of neural activity can be translated across the network to reflect changing input. Importantly, the translation of the activity bumps in CAN models is typically achieved via a velocity-dependent input, reflecting the speed and the direction of the animal’s motion in the physical environment. By summing such velocity inputs over time, one can keep track of position in the environment, in a process called path integration, which many organisms, and grid cells in particular, are capable of (68–72).

CAN models do not provide the only theoretical account of grid cell formation. Alternative models have shown that gridlike firing may also be generated in the absence of CAN dynamics by a combination of spatially tuned input, synaptic plasticity, and cell-intrinsic mechanisms (73–80). A full discussion of grid cell models is beyond the scope of this review (see, e.g., Refs. 53, 81–84), in which we focus on local excitatory and inhibitory microcircuits in the mature animal and the extent to which they exhibit connectivity consistent with CAN dynamics. Although there is a large body of work on spatial coding and microcircuits in the MEC or its functional analogs in species ranging from the fly (e.g., Ref. 88) to humans, the focus of the present review is on the rodent MEC (but see clinical highlights for some insights related to humans).

## 2. FUNCTIONALLY DEFINED CELL TYPES

**FIGURE 2.**
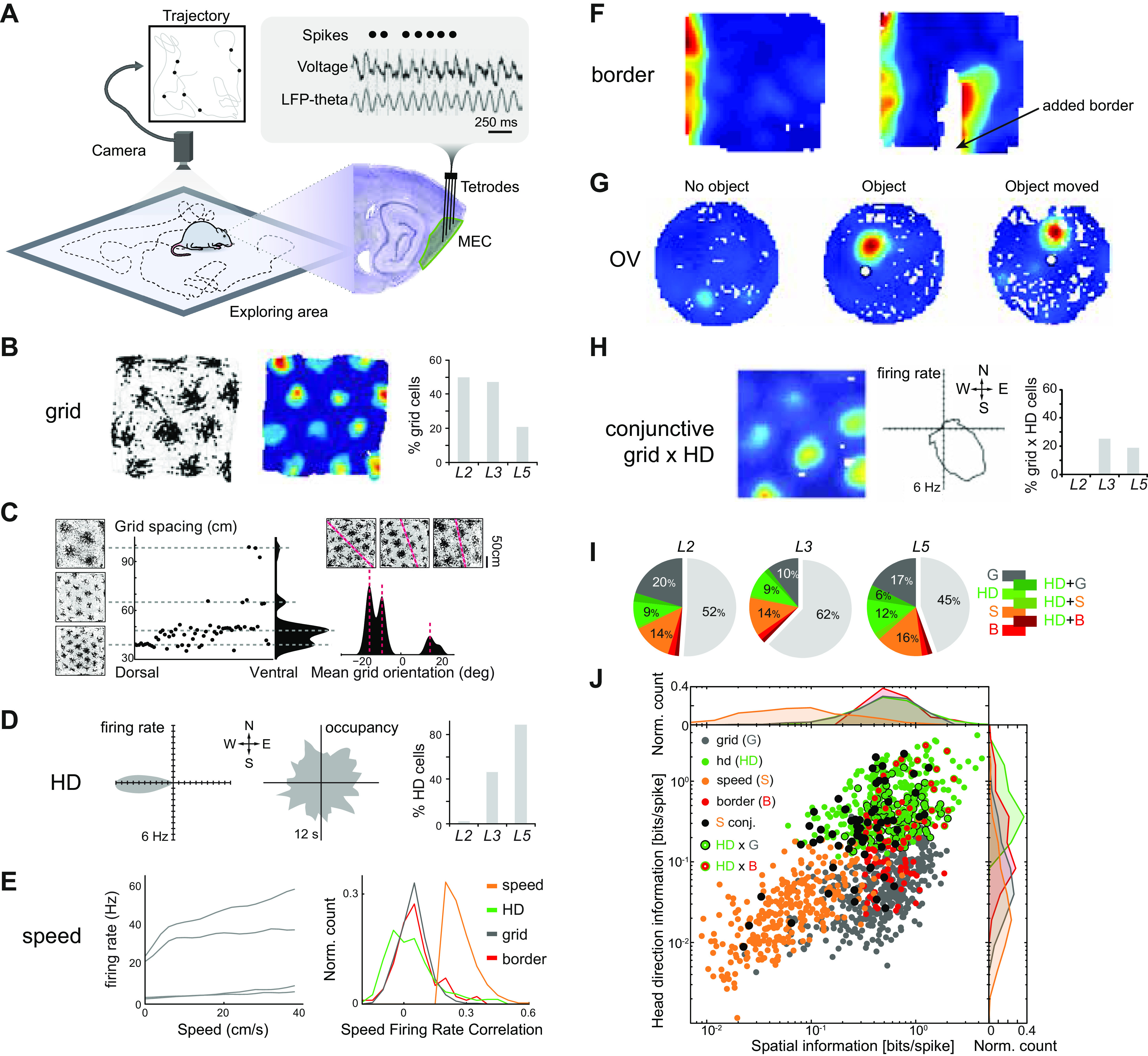
Spatial-coding cells in the superficial medial entorhinal cortex (MEC). *A*: the firing map of a grid cell (*left*; gray lines, rat trajectory; black dots, location at which recorded cell fired a spike). The grid pattern can be more clearly seen in the rate map (*center*; in this panel and all subsequent similar panels, blue colors represent low firing rates, red high). In the three main layers [layers 2 (L2), 3 (L3), 5 (L5)] of MEC containing principal cells, a large percentage of all recorded neurons consists of grid cells (*right*). *B*: extracellular recordings are performed with tetrodes in the MEC (green) as a rat explores a circumscribed area (blue square). Spike times (Spikes) for single isolated units are isolated from the recorded voltage traces (Voltage), which can also be filtered to identify theta oscillations of the local field potential (LFP-theta). Video tracking of the rat’s trajectory (gray curve) is combined with the recorded spike times (black dots) to form a firing map (as in *A*). *C*: the population of grid cells in MEC forms modules displaying discrete values of grid spacing (*left*) and orientation (*right*). Leftmost graph shows grid spacing of single grid cells (black dots) with 3 example firing maps on the *left* and the probability density plotted on the *right* (black). Note that the modules have a dorsoventral organization, with only 1 module being recorded dorsally in this example and 4 ventrally. Rightmost graph shows probability density (*bottom*) of grid orientation for another example recording, with firing maps from 3 example grid cells (*top*) showing the different orientations with the highest probability (red dashed lines). *D*: head direction cell (HD) with firing rate shown in a polar plot as a function of the rat’s head direction (*left*; maximum rate 6 Hz), recorded while the rat oriented its head in all directions over the course of a session (*center*). Percentage of recorded cells with HD tuning (*right*) was smallest in L2. *E*: firing rate of 4 example speed cells as a function of the animal’s speed (*left*) shows both linear and saturating tuning curves. The correlation between speed and firing rate (*right*) is low for most spatially modulated cells (HD, grid, border), suggesting that speed cells (speed) are mostly a separate class of cells. *F*: border cell rate map shows preferential firing at 1 border of the arena (*left*) and additional firing when an extra wall is introduced (*right*; added border) parallel to the preferred border. *G*: object-vector (OV) cell with very low firing rate in absence of an object (*left*) fires at a specific distance and angle from an introduced object (*center* and *right*), independent of the precise location of the object. *H*: conjunctive grid-by-head direction (grid × HD) cell rate map (*left*), firing rate as a function of HD (*center*), and percentages of cells in layers of MEC (*right*). *I*: percentages of all recorded cells in L2, L3, and L5 classified as grid (G), head direction (HD), speed (S), border (B), or conjunctive cells (HD + G, HD + S, HD + B). Note that about half of all cells could not be classified (light gray slice in pie charts); for clarity, only percentages >5% are labeled. OV cells are not included but would account for an additional 15% in superficial layers; their presence in deep layers is not known. *J*: functional cell types cluster in terms of HD and spatial information. Note also presence of conjunctive cells. *A*, *left* and *center*, adapted from Hafting et al. (93) with permission from *Nature*. *A*, *D*, and *H*, right, adapted from Boccara et al. (155) with permission from *Nature Neuroscience*. Traces in *B* adapted from Hafting et al. (185) with permission from *Nature*. The brain in *B* and all panels in *C* are adapted from Stensola et al. (136) with permission from *Nature*. *D* and *H*, *left* and *center*, adapted from Sargolini et al. (153) with permission from *Science*. *E*, *I*, and *J* adapted from Kropff et al. (135) with permission from *Nature*. *F* reprinted from Solstad et al. (186) with permission from *Science*. G reprinted with permission from Høydal et al. (187) with permission from *Nature*.

Extracellular recordings from the MEC have revealed striking correlations between the timing of recorded action potentials (or “spikes”) and variables related to the animal’s behavior. By assigning detected spikes to single neurons and correlating their spike times to particular dimensions of behavior such as location, head direction, or speed, spatially tuned neurons can be identified (FIGURE 2*B*). In most other higher-order brain areas, individual neurons are tuned to a wide array of parameters, displaying mixed selectivity (89). The MEC stands out in the sense that it contains a large number of cells tuned primarily to a single variable. Such functional “cell types” provide useful conceptual building blocks for understanding spatial function (53, 54, 90–92), and their existence has greatly facilitated the search for mechanisms of computation at the network level.

The recorded spike patterns themselves can also be informative regarding the mechanisms underlying spatial coding. Rhythmic firing at a particular frequency [particularly theta frequency, 6–10 Hz (93–95)] and firing bursts versus single spikes (96–98) have both been associated with spatial coding. The spiking patterns of recorded neurons, particularly when combined with spike shapes, can also be used to predict the underlying anatomical cell type, allowing a putative identification of excitatory principal cells and inhibitory interneurons in the MEC (99). Because the population of interneurons is very heterogeneous (discussed below), this identification is likely only correct for those subtypes of interneuron whose firing is most different from principal cells (so-called fast-spiking interneurons, forming <10% of MEC neurons). Other methods such as the cross-correlation between recorded pairs of units [showing, e.g., consistent inhibition in *cell B* when *cell A* fires (100, 101)] or optogenetic tagging (102) can further aid identification.

Neuronal firing patterns also tend to be linked to oscillations of the local field potential (LFP) in specific frequency bands including theta (6–10 Hz), gamma (30–80 Hz), and ripple (120–250 Hz) oscillations, which reflect temporal organization of neuronal activity and correlate with particular brain states (103, 104). Indeed, many recorded cells in the MEC are modulated by such oscillations (105–112). In particular, theta oscillations, which are prominent during active explorative behavior in a wide range of species including rodents and primates, have been proposed to play a role in spatial coding (6, 113–117). In the hippocampus, specific frequency and phase preferences of recorded neurons have been linked to anatomically defined interneuron subtypes (118–124). For the MEC, although intracellular and juxtacellular recordings allowing anatomical identification of neurons have been made in anesthetized and awake rodents (95, 108, 110, 125–134), the relationship between anatomical subtypes and spike timing relative to oscillations remains unclear, so that in most cases extracellularly recorded spike times only allow putative classification of fast-spiking interneurons and principal cells. The great majority of studies have focused on principal cells, since very few (0.3%) grid, HD, and border cells are classified as fast-spiking interneurons, with only speed cells including a significant proportion (25%) of such interneurons (135).

### 2.1. Grid Cells

The grid cell is the most-studied spatially selective cell type in the MEC. Grid cells are characterized by multiple firing fields that are arranged in a periodic manner, forming a hexagonal grid of firing fields (FIGURE 2*A*). This grid is defined by its orientation relative to the environment, spatial phase (location), period (distance between fields), and skewness (shearing-induced asymmetry), and the size and firing rates of its firing fields (93, 136, 137). Given the fact that the grid spatial period changes along the dorsoventral extent of the MEC (93, 94, 138) and different scales cannot coexist within the same CAN, it was predicted that several separate CAN modules should exist (71, 139). The same paper also predicted that inputs from multiple such modules may generate nonperiodic place fields in the hippocampus and that “remapping” of place fields (140, 141) could be driven by changes in the inputs from different grid modules (142, 143). Indeed, subsequent simultaneous recordings from a large number of grid cells confirmed that not only the spatial period but also the orientation and skewness tend to be limited to a few relatively sharp ranges of values (FIGURE 2*C*) (136). Cells with similar values are located in overlapping mediolaterally extended bands, forming at least four functionally independent modules along the dorsoventral axis of the MEC (136). Grid cells within such modules fire at different spatial phases (i.e., at different locations), so that as a population they uniformly cover the entire space.

Interestingly, changing the size and shape of an animal’s environment causes rescaling of the grid pattern (144), as predicted for two-dimensional CANs (145). This rescaling is different for each module and coherent across all cells within a module (136). In conditions where the spatial phase (i.e., firing field locations) of single grid cells changes over time, or the properties of the grid itself change, as happens in altered or novel environments (144, 146), the entire population appears to change coherently (93, 143, 147). Although this tight coupling among grid cells may limit the representational capacity of a module, it makes the behavior very robust to perturbations: the network within a module behaves as a two-dimensional manifold, similar to a continuous attractor pulling the network to the same sets of states that lie along this manifold. In this respect, grid cells differ from hippocampal place cells, whose firing is more high dimensional, with patterns of active cells differing strongly depending on the particular environment or variables associated with it. During novelty, which causes hippocampal remapping, the grid cell network remains stable (147), suggesting that the dynamics of the network do not depend on input from the hippocampus. Indeed, after hippocampal inactivation that strongly reduces grid firing (148), grid cells remain coupled (149), consistent with a model in which the hippocampus provides the main excitatory drive to a CAN where connectivity is purely inhibitory (47, 148). The robust coupling of grid cells within a module persists also in the absence of visual input (62, 93, 150) and during sleep (151, 152), suggesting that the coupling is not dependent on shared sensory inputs but rather reflects local CAN dynamics.

Grid cells have been found in all principal cell layers of the MEC (FIGURE 2*A*), but higher proportions exist superficially particularly in layer 2 (L2) (153). Grid cells in the superficial layers of MEC project to the hippocampus along with HD and border cells (154). Finally, grid cells have also been recorded, together with HD, border, and speed-tuned cells, in the pre- and parasubiculum (155), two areas that provide a large proportion of overall input to the MEC (FIGURE 1*B*) with axons forming layer-specific bands in the superficial MEC (40, 41, 156–159). It is therefore in theory also possible that a specific pattern of inputs from these areas to different modules could explain grid cell firing in the MEC, without any role for local MEC microcircuits. The connectivity in both the presubiculum (160) and parasubiculum (161), together with the availability of HD and speed information in these areas, suggests that grid cell firing could be locally generated there with CAN dynamics. Since connections from the superficial MEC back to the pre- and parasubiculum have been reported (40, 41, 162–166), grid cell firing in the pre- and parasubiculum could also be driven by the MEC. These possibilities remain to be investigated.

### 2.2. Head Direction Cells

First discovered in the presubiculum, head direction cells are defined by their selective firing whenever the animal’s head is facing a particular direction relative to its environment (FIGURE 2*D*) (167). In the rat superficial MEC, HD cells appear to be particularly abundant in layer 3 (L3) and almost absent in L2 (FIGURE 2*D*) (153, 155, 168). In mice, HD tuning is less restricted to L3 (168, 169). In general, layer boundaries are imprecise, and therefore observed percentages of functional cell types within layers can vary across studies (compare FIGURE 2*A* and 2*D* vs. 2*I*; see also, e.g., Refs. 130, 131).

The HD signal is likely generated subcortically, primarily based on vestibular cues (170) in a process that is well modeled by CAN dynamics with head angular velocity inputs, “anchored” to landmarks in the environment (171–174). It has been shown that HD signals propagate via anterior thalamic nuclei and presubiculum to the MEC (175–177), and HD cell activity in both anterior thalamus and presubiculum has a ring topology as predicted by CAN models (see appendix) (178–180). Other thalamic nuclei and the parasubiculum also contain HD-tuned cells and may play similar roles (155, 181–183). Anterior thalamic lesion and inactivation experiments suggest that this HD input is crucial to MEC grid and HD cell firing (184). HD input to grid cells may also be directly observed in conjunctive cells, which have both grid firing and HD tuning (153) (discussed below), and can even be “unmasked” in nonconjunctive grid cells when excitatory drive from the hippocampus is reduced (148). One function of HD cells in the MEC may therefore be to provide grid cells with information regarding the direction of the animal’s movement, consistent with CAN models that require a velocity input (velocity consists of speed and direction) to move the bumps of activity such that the same cells are always active at the same location, regardless of how the animal is moving. However, extrinsic HD inputs could also perform this function. Indeed, a recent experiment showed a dissociation between HD cells and grid cells in the MEC: when grid cell firing in the MEC was affected by changing the shapes of the environment, HD cells in the MEC did not change in a correlated manner, taking much longer than grid cells to reorganize their firing patterns in response to the environmental manipulations (188, 189).

HD cell tuning is influenced by landmarks that the animal uses to orient itself. This is classically shown by putting animals in an environment with a single cue card as a salient (visual) landmark: shifting this cue card by a certain angle usually causes the HD system to also reorient itself, with most HD cells also shifting their preferred head direction (190, 191). In the MEC, it was recently reported that not all HD cells are similarly “visually driven”: when the visual pattern of LEDs on the recording environment walls was changed, HD cells with non-theta-rhythmic firing changed both the preferred direction and strength of their HD tuning, whereas this was not the case for theta-rhythmic HD cells (192). On the other hand, theta-rhythmic HD cells appeared to be much more constrained by CAN dynamics, similar to grid cells (192). It is tempting to speculate that theta-rhythmic HD cells may be part of the same CAN microcircuit as grid cells, which also tend to be theta-rhythmic and also tend to be more affected by the animal’s motion than by visual input (71, 93, 193). Consistent with this idea, optogenetic stimulation of local principal cells (L2 pyramidal cells) (65) recently showed that only a subset of HD cells (defined by their broad HD tuning curves) altered their firing rates, as did grid cells. In contrast, sharply tuned HD cells did not alter their firing rates, suggesting that whereas grid cells and broadly tuned HD cells are part of the same microcircuit (together with interneurons), the sharply tuned HD cells are not. Interestingly, most sharply tuned HD cells in this study were also non-theta-rhythmic (Ipshita Zutshi and Stefan Leutgeb, personal communication), suggesting that they may overlap with the non-theta-rhythmic visually driven HD cells in the Kornienko et al. study (192), forming a single population of HD cells that may not be part of the CAN underlying grid cell firing (194). In contrast, the more broadly tuned and theta-rhythmic HD cells would be predicted to be more connected to the CAN underlying grid cell firing. These broadly tuned HD cells may encode the direction of prospective trajectories encoded by grid cell ensembles during so-called theta sequences, which would explain why their firing is not as tightly correlated to ongoing head direction (194).

Finally, it should be pointed out that the animal’s head direction is not the same as the animal’s movement direction: an animal’s head is often facing in a different direction as it is moving (195). In fact, it has been suggested that, in contrast to movement direction input, HD input cannot sustain stable grid firing as an animal moves around its environment (195). It remains to be determined whether, and under which circumstances, the MEC *1*) receives explicit input on the animal’s movement direction from other areas, for instance the medial septum (196); *2*) computes location without any use of movement direction, for instance based on spatial input from hippocampus (80); or *3*) computes movement direction and/or location based on sensory input or self-motion (proprioceptive or motor) signals (197, 198). Interestingly, to detect movement direction based on auditory, visual, or somatosensory (e.g., whisker) cues, HD cells could theoretically play a role in transferring a head-centered frame of reference to an environment-linked reference frame.

### 2.3. Speed Cells

Speed information in the MEC is present in the firing patterns of some grid, border, and HD cells, as well as in specific “speed cells” (FIGURE 2*E*), which include fast-spiking parvalbumin-expressing (i.e., inhibitory) cells projecting to the hippocampus (61, 62, 135, 153, 199, 200). In fact, speed-sensitive cells have been reported in many cortical and subcortical areas (201–208), perhaps reflecting the fact that speed can be easily computed in the brain based on self-motion (motor), visual (optic flow), or other sensory information (e.g., vestibular). Together with HD cells, speed cells can inform the grid CAN about the animal’s current velocity; as mentioned above, this information is crucial for spatial coding since it enables the firing fields of the grid to remain locked to the same locations, regardless of how the animal moves.

The precise mechanisms of how speed and HD information are combined remain unclear, but several studies have suggested that speed cells may be more closely linked to grid cell firing than HD cells (189, 193, 209). Similar to grid and HD cells, the firing of speed cells is mostly context independent (135), although some changes in the speed code have been reported, for instance in darkness (62, 150, 210), when the shape or size of the environment is changed (189), or even when a visual pattern is changed along a linear track (62). The finding that changes in speed cell firing tend to be correlated with changes in grid cell coding suggests that speed cells may be embedded in functional grid cell modules, possibly providing part of the velocity input needed to shift activity bumps in CAN models.

Two recent findings appear to challenge this idea. First, a nonlinear relationship between firing rate and speed has been reported under some circumstances (FIGURE 2*E*), which could make it difficult to use firing rate as a simple readout for speed (211). However, it remains to be seen whether such a nonlinearity, which is mostly related to slow speeds and may be explained by more general state changes (135, 212), is limiting to the computation of grid firing. It is worth noting that above a minimum activation threshold speed and firing rate are linearly correlated over a wide range of running speeds, in agreement with a possible role for speed cells in path integration (135, 212). Second, it has been argued that the reliable “readout” of a speed cell’s firing rate may take several seconds, and that this would be too long to be used as an online measure of speed (213). Although this may be true for the readout of single cells, readout could be much faster when based on large populations of cells, where readout likely takes place. This is particularly evident if the population also includes the subset of highly modulated speed cells with high firing rates and interneuron-like properties (which were in fact detected).

### 2.4. Border Cells

Border cells (FIGURE 2*F*) fire selectively when the animal is at a certain position relative to a geometric “border” of its environment (186, 214). The border signal is also present upstream in the presubiculum (155), where the integration of a pure HD signal (e.g., north) from anterior thalamus with local egocentric information (e.g., wall on the animal’s left) may lead to spatial information [the wall is in the west in this example (215)]. Similar merging of egocentric and HD information may also take place in the MEC itself, based on direct inputs from the postrhinal cortex (216–218), which was recently shown to contain a population of egocentric border cells (54, 219). In the context of path-integrating CAN networks, border cells could help to “anchor” the representation to the environment, particularly for elements in the environment that can block the animal’s trajectory (187). Periodic anchoring of the CAN to the environment is necessary because path integration mechanisms generally accumulate random errors over time, and this is also true for grid cells: they coherently accrue “drift” as the mouse moves away from the boundaries of the environment, and this drift is “reset” whenever the mouse encounters a boundary in a manner consistent with border cell input to a CAN grid model (139, 220).

### 2.5. Object-Vector Cells

Object-vector (OV) cells (FIGURE 2*G*) fire whenever the animal is at a particular distance and direction (i.e., a vector) from a particular “object” in the environment (187), in a manner that appears independent of the precise properties of the object. Across environments, the orientation of the object-vector can rotate, but the distance metric remains the same, and pairs of cells keep their relative orientations, also with respect to simultaneously recorded grid and HD cells (187). Although the latter finding is only based on a small sample, and for example the two kinds of HD cells described above were not differentiated, it suggests that OV and grid cells may be part of a single low-dimensional CAN for the representation of location. OV cells have thus far only been described in the superficial MEC, where they make up ∼15% of all recorded cells (187). They may have a role similar to border cells, in the sense that they could help to link grid cell firing to particular landmarks or discrete sensory features of the environment. Recently described “cue cells,” which fire near visual landmarks across different virtual reality environments, have also been posited to belong to a similar population as OV cells (221). Although the trajectory of the mice in these experiments was along a one-dimensional virtual track, with little opportunity to infer directional tuning as would be expected for OV cells, the cue cells did show a preference for the left or right side when cues were presented on both sides of the track (221). In real-world recordings, these cells showed irregular but stable firing patterns, suggesting they were neither grid nor border cells, but their responses to real objects remain untested. For an excellent review of a wide range of other recently discovered vector-coding cells, see Ref. 54.

### 2.6. Conjunctive Cells

Conjunctive cells (FIGURE 2, *H*–*J*) encode different combinations of speed, place, and head direction (135, 153, 186). The grid × HD conjunctive cells are the most common (FIGURE 2, *H* and *I*) and have been studied the most. HD coding of grid cells appears to be somewhat bimodal, with tuning being either very sharp or very wide, rather than forming a clear continuum (222), and may be present in all principal cell layers (FIGURE 2*I*) (135), although reported proportions differ greatly (135, 153, 186). Consistent with a crucial role for conjunctive “integrator” cells in path integration (71, 145, 223), it has been proposed that deep-layer conjunctive cells integrate velocity inputs to generate grid cell firing, in turn providing feedforward input to superficial pure grid cells (222, 224–226).

It has been suggested that most cells in the MEC are conjunctive, in the sense that their firing exhibits some degree of tuning by all three variables, i.e., place, speed, and HD (227). However, these variables tend to be unequally weighted, and most cells in the MEC display a strong preference for one particular kind of spatial coding, rather than being truly conjunctive (FIGURE 2, *I* and *J*). Thus, perhaps more than in any other well-investigated high-level cortical region, functionally defined cell types describe an important property of neural organization in the MEC.

## 3. ANATOMICALLY DEFINED CELL TYPES

**FIGURE 3.**
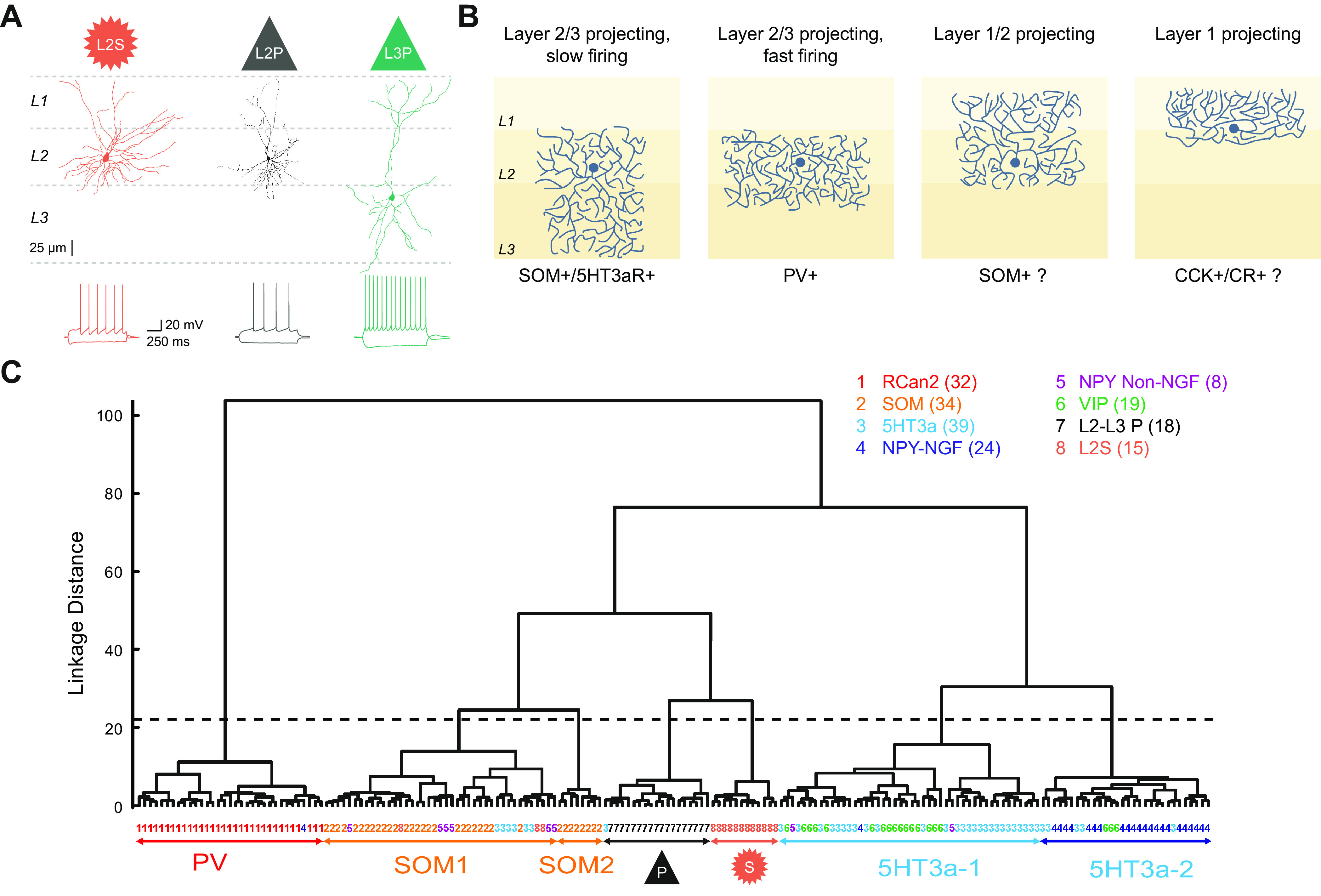
Anatomically, electrophysiologically, and molecularly defined cell types within superficial layers of the medial entorhinal cortex (MEC). *A*: reconstructed somata and dendrites (*top*) and voltage traces (*bottom*; overlaid responses to current injection steps) for the main excitatory cell types: L2S, layer 2 (L2) stellate cell; L2P, L2 pyramidal cell; L3P, layer 3 (L3) pyramidal cell. Note the presence of a sag potential at hyperpolarized voltages for the L2S cell. *B*: schematic representation of axonal projections for 4 interneuron classes in the superficial MEC, classified via clustering based on intrinsic electrophysiological and axonal projection data from glutamate decarboxylase 2 (GAD2)+ neurons. Note that apart from the fast-firing group (2nd panel), cell classes identified in this study only partially coincide with common genetic markers (shown below each panel). CCK, cholecystokinin; CR, calretinin; PV, parvalbumin; SOM, somatostatin; 5HT3aR, serotonin receptor type 3a. *C*: hierarchical clustering based on 9 electrophysiological parameters suggests that 8 cell types based on anatomical and genetic criteria (listed *top right*) can be grouped (groups shown with arrows at *bottom*; based on a cutoff linkage distance shown as a dashed line) into 2 principal cell types (P for L2P and L3P, S for L2S) and 5 interneuron types, which we named PV, SOM1, SOM2, 5HT3a-1, and 5HT3a-2. Linkage distance is a measure of similarity between cells, which are shown as colored numbers at *bottom* (cells linked at a smaller linkage distance are more similar). Note that the groups only partially coincide with the anatomical/genetic types [listed at *top*, number of recorded cells in parentheses; RCan2, cells in a regulator of calcineurin 2 mouse line used here to identify PV+ fast-spiking (FS) interneurons; SOM, subpopulation of SOM+ cells from GIN mouse line; NPY-NGF, cells anatomically identified as neurogliaform (NGF) cells in a neuropeptide Y mouse line; NPY Non-NGF, cells anatomically identified as non-NGF cells in a neuropeptide Y mouse line; VIP, cells in vasointestinal protein mouse line; P, pyramidal cells; L2S were identified by soma size and shape]. *B* adapted from Martínez et al. (232) with permission from *eNeuro*. *C* adapted from Ferrante et al. (233) with permission from *Cerebral Cortex*.

To understand the microcircuits potentially underlying the spatial coding properties in MEC neurons outlined above, it is important to know the elements that make up these circuits. Layer 2 of the MEC is populated by two main types of excitatory cells, namely the stellate cell (L2S) and the pyramidal cell (L2P), which can be differentiated on the basis of their morphologies and physiological properties (FIGURE 3, *A* and *C*), projection targets (FIGURE 1*B*), and molecular profiles (58, 228–231). A recent study also defined “intermediate stellate” (intS) and “intermediate pyramidal” (intP) cells, based on morphological and electrophysiological criteria, which partly coexpressed pyramidal and stellate cell markers (50). However, these intermediate cell types could not be identified in another data set (52). Layer 3 is only populated by pyramidal cells (L3P), with characteristic intrinsic properties (234). Both layers additionally contain a very heterogeneous minority of ∼10% gamma-aminobutyric acid (GABA)-releasing inhibitory interneurons, which mostly have local axonal projections (FIGURE 3*B*) but also include a minority of GABAergic cells with projections to the hippocampus (199, 235).

### 3.1. Layer 2 Stellate Cells

L2S cells make up ∼50–75% of principal cells in MEC L2 (228, 236, 237). L2Ss were first described by Ramon y Cajal, who called them “cellulas estrelladas grandes,” or large starlike cells, reflecting their relatively large, polygonal soma emitting four or five dendrites (FIGURE 3*A*) (229, 238). They provide the main output from MEC to the ipsilateral dentate gyrus of the hippocampus (FIGURE 1*B*) (85), express reelin (58), and show intrinsic membrane properties that are markedly different from L2P cells: L2Ss are characterized by subthreshold membrane potential oscillations, membrane potential resonance, clustered firing, and a hyperpolarization-activated cation current *I*_h_ that causes a depolarizing “sag” (FIGURE 3*A*) in response to hyperpolarizing current steps (228, 230, 239–241).

Synaptic integration in stellate neurons has been investigated extensively (242, 243), and the width of their temporal integration windows and excitatory postsynaptic potentials (EPSPs) correlates with grid spacing along the dorsoventral axis of the MEC: broader temporal integration windows and EPSPs at more ventral levels may help to explain the increased grid spacing in ventral MEC (93, 94, 138). It has been shown that prolonged synaptic currents can indeed lead to larger grid field sizes and spacing in a CAN model of grid firing (244, 245). Similarly, slower intrinsic subthreshold oscillation frequency in ventral MEC stellates has been proposed to underlie this spacing gradient (240). HCN1 channels are likely to play an important role in defining the scale of spatial tuning (241, 246, 247) by mediating *I*_h_ (together with HCN2). Interestingly, a dorsoventral grid spacing gradient persists after knockout of HCN1 (248), suggesting that other factors must also be considered. However, in the latter study grid spacing and field size were expanded at all dorsoventral levels, together with a drop in the frequency of the accompanying theta-rhythmic firing in stellate cells, particularly at higher speeds; this suggests a role for *I*_h_ in determining the gain of the speed-dependent input in stellate cells, which moves around activity bumps in a CAN. However, feedforward models of the development of grid cell patterns also predict a tight relation between adaptation currents and the scale of spatial tuning (74, 80).

L2S cells can be grid cells but also head direction, speed, border, or conjunctive cells (67, 127, 249, 250). This lack of 1:1 correspondence between L2S cells and any type of functional cell is not an exceptional trait of L2S cells but seems to be a general principle in the MEC, although of course more fine-grained classifications may yet uncover specific molecular markers, morphological traits, or intrinsic electrophysiological properties that correspond to a particular functional cell class.

### 3.2. Layer 2 Pyramidal Cells

L2P cells are the second excitatory cell type in L2 of the MEC, making up 25–40% of principal cells in this layer (228, 236, 237). Like L2S cells, their dendrites are mostly limited to layers 1 and 2, but in contrast to L2S cells, they display a pyramidal cell body shape with a clear apical dendrite (FIGURE 3*A*). L2Ps can be further identified by immunohistochemical markers like calbindin or wfs1 (58, 87, 129). They are organized in a hexagonal pattern of patches or “islands” arranged in a skewed gridlike manner, with their apical dendrites bundled together in layer 1 (87, 129, 251, 252). These patches may be part of a more elaborate structural organization that appears to be present in the MEC of many species that includes also patches identified by zinc, bundling of dendrites from deeper layers, and patch-specific inputs (237, 253). Note that similar structures have also been identified in many other cortical regions and may therefore reflect a more general cortical organizational principle (254–257).

L2Ps comprise a heterogeneous population in terms of their axonal projections, with ∼50% comprising “excitatory interneurons,” with local but relatively widespread axonal projections restricted to the superficial layers of ipsilateral MEC or lateral entorhinal cortex (LEC), ∼30% projecting to the contralateral EC, 20% to the hippocampal area CA1, and 2% to medial septum [as recently quantified in rats (60); see also FIGURE 1*B* (50, 58, 65, 87, 258)]. Although it seems clear that most L2P cells have local axon collaterals, and at least some of these also project to the contralateral EC (60, 95), the extent to which the same cells project to multiple distal targets is still unclear. Recent advances in whole brain microscopy, genetic cell type-specific labeling, and tissue clearing have made it possible to create whole brain reconstructions of relatively large numbers of neurons and their long-range arborizations (259, 260), but this approach has not yet been applied to cells in the MEC.

Although L2P intrinsic membrane properties are not conducive to the generation of theta oscillations (58), and they do not display a prominent sag potential (FIGURE 3*A*), it has been reported that in vivo L2P cells in fact have higher theta rhythmicity than stellate cells (97, 130; but see also 126). A possible source of this theta rhythmicity could be input from the medial septum (261). Besides GABAergic and glutamatergic inputs likely to be important for driving theta oscillations (205, 206, 262–264), this input also includes cholinergic fibers that may modulate theta rhythmicity (265–266) and specifically affect pyramidal patches (129). Alternatively, or in addition, theta rhythmicity in L2P cells may also be driven by the observed patch-specific axonal projections from the parasubiculum (181), a region with strong theta rhythmicity (155, 267). Like L2S cells, L2P cells can also have a wide range of functional coding properties (96, 127, 130, 249), including grid firing, although determining the precise proportions may require methods that allow tagging of specific subpopulations of functionally characterized L2P cells, such as two-photon calcium imaging microscopy in freely moving animals (268, 269).

### 3.3. Layer 3 Pyramidal Cells

Layer 3 of the MEC is one of the widest layers. It is populated by L3P cells expressing the oxidation resistance 1 (Oxr1) gene (270) that project bilaterally to CA1 and the subiculum, as well as providing the large majority of projections to the contralateral MEC (FIGURE 1*B*) (43, 60, 85, 271, 272). L3P dendrites are largely limited to superficial layers 1–3 (FIGURE 3*A*) and appear to avoid L2P patches in L2 (131), leading to the suggestion that L2P cells may receive preferential inputs from the parasubiculum (181) compared with L2S and L3P cells (97).

L3P cells appear to form a largely homogeneous population in terms of intrinsic electrophysiological properties, regardless of whether they project to the ipsilateral hippocampus or to the contralateral side (131). Many L3P cells respond to LEC stimulation with EPSPs lasting >3 s (273); slow hyperpolarization lasting up to tens of seconds has also been reported (234, 271, 272). Consistent with these properties, L3Ps appear to preferentially respond to low-frequency inputs, in contrast to L2Ss, which are more tuned to higher-frequency input (273). Part of the input to L3Ps is coming from deep layers, which likely mediate input from the hippocampus (45, 273–275).

L3P cells were also shown to have a high excitability in vivo (108) and fire spontaneously even in vitro, likely driven by a persistent Na^+^ conductance (271). In contrast to L2S cells, L3Ps do not display subthreshold theta-frequency membrane oscillations in vitro (108) or in vivo (108). However, both L3P and L2S cells seem to be relatively weakly phase-locked to the LFP theta in comparison to L2P cells (97). Earlier extracellular recordings have also shown that L3P cells are less theta-phase-locked than L2 principal cells, which could not be further differentiated (108, 112, 276).

### 3.4. Inhibitory Interneurons

For a long time, it has been clear that principal cells in the superficial MEC receive strong inhibitory input, both via spontaneously released GABA (277, 278) and via action potential-driven GABA release (234, 272, 273, 279). The latter can both mediate feedforward inhibition, as shown via electrical stimulation of the deep layers of the MEC, the subiculum, or parasubiculum (272, 273, 279, 280), or provide feedback inhibition, as suggested by local stimulation experiments and paired recordings (13, 46, 47, 58, 281). Potentially, all three types of inhibition could play a role in spatial coding, but particularly feedback inhibition is expected to play a role in CAN dynamics. In various models, inhibitory interneurons are either the exclusive mediator of connections between excitatory principal cells or work in concert with recurrent excitatory connections to generate a “Mexican hat”-type connectivity profile, in which excitatory cells excite nearby cells but inhibit cells further away (47, 48, 139, 282). One important issue to deal with when searching for the anatomical substrate of inhibition as specified, e.g., in particular CAN models, is that inhibitory interneurons in the cortex, though forming only ∼10–20% of all neurons, are a very heterogeneous population. They can be divided into three generally nonoverlapping classes expressing parvalbumin (PV+), somatostatin (SOM+), or the serotonin receptor type 3a (5HT3R+) (50, 64, 283–289).

The strong inhibition recorded electrophysiologically within superficial layer principal cells coincides with a dense band of GABAergic fibers (290), later shown to consist mostly of axons from PV+ basket cells (46, 291). By specifically innervating somata, basket cells are able to exert strong control over the output of their postsynaptic cells, which include both inhibitory and excitatory cells in superficial MEC (13, 47, 48, 50, 58, 292). PV+ basket cells fire at high rates in vivo and largely coincide with “fast-spiking” (FS) interneurons. Within layer 2, single PV+ basket cell axons contact the somata of both L2P and L2S cells, although L2P cells have ∼40% more PV+ perisomatic boutons (13). In addition to PV+ basket cells, the superficial MEC also contains a much lower number of axo-axonic cells (293), also known as chandelier cells. These cells, presumably also expressing PV as they do in other brain areas (291, 294), exert strong control over postsynaptic excitatory cells through specifically targeting the axon-initial segments of principal cells (295). Both types of PV+ cells have very divergent connectivity. Together with their high firing rate and the fact that they make up ∼50% of all GABAergic interneurons in the MEC (296), this makes PV+ interneurons potentially very important in terms of controlling the much larger population of principal cells, albeit in a likely nonspecific manner.

SOM+ interneurons are specialized in providing inhibition to postsynaptic dendrites (297), rather than to somata or axons. Thus, they are more likely to influence dendritic integration of inputs, rather than directly controlling the action potential output of their target cells. In the neocortex, these cells form a diverse group, with subtypes differing in terms of morphology and firing properties in awake mice, which have been related to differences in innervation by other interneurons and in modulation by the neuromodulator acetylcholine (298). SOM+ cells partly coincide with low-threshold spiking neurons and tend to fire at lower rates than FS cells. They also tend to display synaptic facilitation, in contrast to PV+ cells, making them more likely to respond to sustained inputs (within a certain time frame) rather than encoding precise onsets. Finally, by modulation of dendritic spiking and plasticity in postsynaptic pyramidal cells, SOM+ cells have been implicated in the generation of spike bursts and place field firing in the hippocampus (299–301). This could be relevant for understanding spatial coding in MEC, given the prevalence of burst firing among grid cells (96) and evidence for dendritic spiking in both L2P and L2S cells (128).

5HT3aR+ interneurons are perhaps the most heterogeneous group (302). This group includes interneurons expressing calretinin (CR+), neuropeptide Y (NPY+), vasoactive intestinal peptide (VIP+), or cholecystokinin (CCK+). Interestingly, CCK+ basket cells in the MEC inhibit layer 2 principal cells, with a strong preference for L2P somata (13, 58). The greater number of CCK+ punctae onto L2P compared to L2S somata suggests that L2P cells may be more influenced by neuromodulators, since receptors for cannabinoids, serotonin, and acetylcholine are all present on CCK basket cells (303). Other 5HT3aR+ interneurons tend to preferentially target interneurons rather than principal cells; this includes 5HT3aR+ interneurons expressing VIP, which are often found in cortical layer 1, and those expressing CR (304). VIP cells, driven by acetylcholine on a fast timescale via nicotinic receptors, can mediate learned responses to sensory inputs by inhibiting SOM+ cells (305–309). Similar circuits, potentially also involving non-VIP layer 1 interneurons inhibiting PV+ cells (310), are likely to set the level of inhibition in the superficial MEC. Whether this has a role in spatial coding remains a matter of speculation. It has been suggested that VIP cells could disinhibit grid cell firing specifically whenever an animal enters a particular location (233), providing one possible explanation for the depolarizing ramp-up of the membrane potential of stellate cells as they enter a firing field (126, 127). This would imply that VIP cells fire in a gridlike pattern, which is unlikely but cannot be ruled out since most extracellularly recorded “principal cell” populations also include non-FS interneurons such as VIP cells. VIP-mediated disinhibition of MEC border cells, specifically during whisking, has also been posited, based on data from monosynaptic retrograde rabies revealing that VIP cells receive input from the mesencephalic trigeminal nucleus Me5, an area in the brain stem that encodes whisker-related proprioceptive information (311).

Two recent studies used clustering approaches in combination with in vitro patch-clamp recordings from the superficial MEC to see to what extent the molecular markers outlined above correlate to anatomical or electrophysiological parameters. One study used mice expressing Cre under the glutamate decarboxylase 2 (GAD2) promoter, which codes for the protein GAD67, one of the two main markers for GABAergic cells in the cortex, together with a PV-Cre mouse line (232). Based on five electrophysiological and four anatomical parameters, they performed a principal component analysis (PCA) to reduce the data to four orthogonal dimensions that together could explain 80% of the variance; *k*-means clustering was then applied for a range of 2–16 clusters, with optimal results found when the data were divided into four groups of cells with distinct axonal projection patterns (FIGURE 3*B*) and electrophysiological properties. Unfortunately, apart from the PV+ FS group, these groups did not coincide in a simple manner with particular molecular markers, and separate clusters based on either electrophysiological or anatomical parameters only showed a 58% overlap. In a second study, interneurons were recorded from five mouse lines including a PV-like Cre line, a SOM-like Cre line, and 5HT3aR-Cre mice (233). Based on nine recorded electrophysiological parameters for each cell, hierarchical clustering was used to derive five groups of interneurons (FIGURE 3*C*). This functional classification could predict the molecular cell class with 81% accuracy, with only one group (PV+ cells) clearly coinciding with a particular molecular marker, and having similar properties as the PV+ groups defined by Martínez et al. (232) (FIGURE 3*B*). Thus, apart from PV+ FS cells, the best way to group the molecular, morphological, and electrophysiological properties of interneurons in the MEC into separate classes remains unclear.

How are all these anatomical cell types connected to form microcircuits that can generate the spatial coding of the functional cell types described above? This connectivity has recently been investigated in a number of studies using both in vivo and in vitro approaches, which we describe next.

## 4. MICROCIRCUITS

The connectivity between cells in the MEC has mostly been investigated in superficial layers 2 and 3, with paired patch-clamp recordings in slice preparations. This method allows labeling of recorded neurons and, in principle, an unequivocal identification of the anatomical cell types involved and their precise location. Other, more indirect methods have been applied in vivo, including the identification of putative monosynaptic interaction between extracellularly recorded units, often in combination with optogenetic stimulation. The genetic tagging of anatomical populations based on specific promoters has been a key tool for both in vivo and in vitro connectivity studies, enabling the expression of fluorescent proteins, opto- or chemogenetic actuators, and activity sensors in specific anatomical cell types (to the extent that such cell types can be identified with molecular markers).

### 4.1. Excitatory Connections in Superficial Layers

All three anatomical principal cell types in superficial MEC appear to receive the majority of their inputs from superficial rather than deep layers (45), consistent with the fact that these cells do not extend dendrites into the deep layers (FIGURE 3*A*). Thus, the local microcircuit in the superficial MEC, rather than the full column, may already be sufficient for the generation of the spatial coding in these cells. Here, we review the connectivity between these superficial excitatory cells in some detail and discuss the extent to which this may constrain CAN models of spatial coding, particularly grid cell firing. Note that although several possible connectivity schemes may generate a gridlike pattern in a CAN (see appendix), it is still important to see which precise scheme is implemented. Are all anatomically defined cell types part of a single interconnected CAN, or is there one interconnected cell type that generates grid firing that is then propagated to other cell types? Cell types that provide output to many local cells without receiving local input may play a role in translating the activity bumps of a two-dimensional (2-D) CAN sheet, or otherwise modulate how extrinsic inputs affect the circuit, but cannot participate in the generation of the sheet itself.

#### 4.1.1. L2S → L2S.

**FIGURE 4.**
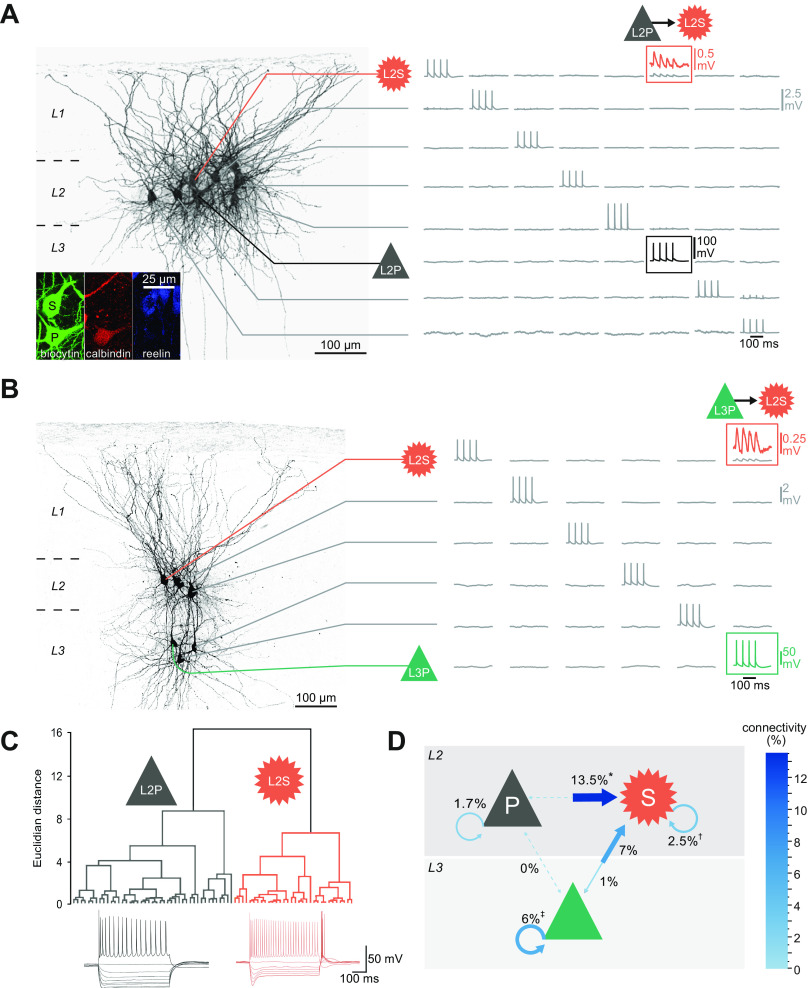
Superficial medial entorhinal cortex (MEC) excitatory microcircuits. *A*: example of 8 simultaneously recorded cells, showing a connection between a presynaptic layer 2 pyramidal (L2P) cell expressing the L2P marker calbindin (*inset*, P) and a postsynaptic layer 2 stellate (L2S) cell expressing reelin (*inset*, S). Columns depict responses of all possible postsynaptic cells to stimulation of 1 cell (stimulation shown along the diagonal). Only 1 cell (the L2S cell in *row* 1) showed a response in this case (red trace on *top*, magnification). *B*: example of 6 simultaneously recorded cells, showing a connection between a presynaptic layer 3 pyramidal (L3P) cell and a postsynaptic L2S cell. *C*: hierarchical classification of principal cells in layer 2; for the 2 main classes, characteristic voltage traces in response to current injection are shown at *bottom*. *D*: summary of excitatory microcircuits. Blue arrows depict connectivity as % of postsynaptic cells showing a response to induced presynaptic spikes. Percentages are from Winterer et al. (52). *Earlier studies reported 0% based on a smaller sample (47) or a different definition of L2P cells (50). †Earlier studies reported 0% (47, 48, 50). ‡An earlier study reported 9% between L3Ps (44). See also TABLE 1. *A* and *B* adapted from Winterer et al. (52) with permission from *Cell Reports*. *C* adapted from Grosser et al. (292) with permission from *eNeuro*.

Direct data on connectivity were recently acquired by performing patch-clamp recordings of up to eight cells simultaneously (52). By stimulating each cell one at a time while detecting responses in all the others, synaptic connections were tested for up to 56 pairs of cells at a time (FIGURE 4, *A* and *B*). With this method, cells could also be electrophysiologically characterized and filled with biocytin, allowing post hoc immunohistochemical analysis: L2S cells were identified by their expression of reelin and relatively long depolarizing “sag” potentials in response to hyperpolarizing voltage steps, whereas L2P cells expressed calbindin and showed shorter sag potentials.

**Table 1.** Reported connectivity of principal cell types in the superficial MEC

Presynaptic	Postsynaptic	Connectivity, %	*n* Pairs Tested	Species/Strain	Cell Identification	Publication
L2S	L2S	0ª	644ª	Long-Evans rat	Intrinsic ephys°; morphology in subset	Couey et al. (47)
** **	L2S	0	56	Thy1-ChR2-YFP mouse	Sag potential, clustered APs	Pastoll et al. (48)
** **	L2S	0	2,200^b^	Thy1-ChR2-YFP mouse	Sag potential, clustered APs	Pastoll et al. (48)
** **	L2S	0	100	Uch1-Cre mouse	Intrinsic ephys^; apical dendrite*	Fuchs et al. (50)
** **	L2S	2.49	882	Wistar rat	Sag potential	Winterer et al. (52)
	L2IntS	4.3	47	Uch1-Cre mouse	Intrinsic ephys^; apical dendrite	Fuchs et al. (50)
	L2P	8.3	24	Long-Evans rat	Intrinsic ephys°; morphology in subset	Couey et al. (47)
	L2P	0	38	CB-Cre mouse	Intrinsic ephys^; apical dendrite	Fuchs et al. (50)
	L2P	0	126	Wistar rat	Sag potential	Winterer et al. (52)
	L2IntP	0	39	CB-Cre mouse	Intrinsic ephys^; apical dendrite	Fuchs et al. (50)
	L3P	1	100	Wistar rat	Sag potential	Winterer et al. (52)
L2IntS	L2S	6.5	46	UCh1-Cre mouse	Intrinsic ephys^; apical dendrite	Fuchs et al. (50)
L2P	L2P	1.8	56	CB-Cre mouse	Intrinsic ephys^; apical dendrite	Fuchs et al. (50)
** **	L2P	1.6	64	Wistar rat	Sag potential	Winterer et al. (52)
	L2IntP	4.8	42	CB-Cre mouse	Intrinsic ephys^; apical dendrite	Fuchs et al. (50)
	L2S	0	38	CB-Cre mouse	Intrinsic ephys^; apical dendrite	Fuchs et al. (50)
	L2S	13.49	126	Wistar rat	Sag potential	Winterer et al. (52)
	L2S	0	52	Long-Evans rat	Intrinsic ephys°; morphology in subset	Couey et al. (47)
	L3P	0	84	Wistar rat	Sag potential	Winterer et al. (52)
L2IntP	L2P	7.5	40	mouse	4 intrinsic ephys parameters; apical dendrite	Fuchs et al. (50)
	L2IntP	4.7	43	mouse	4 intrinsic ephys parameters; apical dendrite	Fuchs et al. (50)
	L2S	10.0	40	mouse	4 intrinsic ephys parameters; apical dendrite	Fuchs et al. (50)
L3P	L3P	8.4	393	Wistar rat	Regular firing	Dhillon and Jones (44)
	L3P	5.7	209	Wistar rat	Input resistance, resting membrane potential	Winterer et al. (52)
	L2P	0	84	Wistar rat	Sag potential	Winterer et al. (52)
	L2S	7.0	100	Wistar rat	Sag potential	Winterer et al. (52)

AP, action potential; ephys, electrophysiology; L2IntP, layer 2 intermediate pyramidal cell; L2IntS, layer 2 intermediate stellate cell; L2P, layer 2 pyramidal cell; L2S, layer 2 stellate cell; L3P, layer 3 pyramidal cell. ^a^Not fully clear from text how many pairs tested under which conditions; sporadic connectivity (0.3–1.8%) seen before p28 and in 3/246 pairs (1.2%) in adults with GABAergic input and Kv1 channels blocked “only in response to multiple simultaneous stimulations.” ^b^Estimated based on 11 cells recorded, each with estimated 200 channelrhodopsin (ChR)-expressing L2S cells stimulated by light. *Not fully clear from text whether dendrites were used for identification of stellate cells. °Sag potential, ratio first and second interspike interval, input resistance, high-frequency burst firing. ^Sag potential, ratio first and second interspike interval, depolarizing afterpotential, latency to first spike.

Based on these criteria, L2S cells received monosynaptic inputs from other L2S cells (2.5%) (52) (FIGURE 4*D*). Although this percentage is low compared to the ∼10% or more typically reported in other cortices (312, 313), this recurrent connectivity (based on 882 recorded pairs of L2Ss) is considerably higher than previously reported: several studies (47, 48, 50) observed 0 connections between L2S cells. The fact that L2S cells were identified in different ways in these four studies may explain part of the discrepancy: if one includes the connectivity between pure and intermediate stellate cells identified in the Fuchs et al. (50) study (4–7%), their overall L2S connectivity becomes consistent with the 2.5% reported by Winterer et al. (52).

Interestingly, even when Couey et al. (47) stimulated up to three stellate cells simultaneously (in clusters of up to 4 simultaneously recorded stellate cells), no response in the fourth stellate cell was observed. This experiment is important because a lack of monosynaptic responses in any paired patch-clamp recording simply means that there is insufficient synaptic input at the dendrites of the postsynaptic cell (within a particular spatiotemporal window) to elicit a response at the soma, but does not necessarily imply a lack of synaptic connections. Stimulating several presynaptic cells increases the chances of detecting such a connection, but it is unclear how much: most neurons receive thousands of synaptic inputs, typically with a lognormal distribution of synaptic strengths such that the majority of synapses are relatively weak (314). In other words, a lack of responses in a paired recording may indicate that many convergent inputs are needed to elicit a somatic response, rather than showing a lack of synaptic connections per se. Such functionally weak synapses can be detected via electron microscopy (EM), and indeed a very high percentage (22%) of connectivity between L2S pairs was reported in a sample of nine EM-reconstructed L2S cells (51). It should, however, be noted that these EM data are from a very small sample, and differences in identifying L2S cells may also have led to an overestimation of connectivity in this particular case: L2S cells were identified based on their larger somata, and theoretically some L2P or intermediate cells (with higher connectivity) may have been misclassified as L2S. Even relatively strong synaptic responses at the dendrites may be suppressed by coincident inhibitory inputs or other dendritic mechanisms, which may shunt excitatory potentials or affect nonlinear mechanisms of propagation along the dendrites (315, 316), again potentially leading to an underesimate of connectivity when measuring at the soma. Of course, what is more important for the network (anatomically identified synapses with locally restricted effects or functional connections affecting the neuron’s somatic membrane potential) depends on the particular question.

There are two more reasons that paired patch-clamp recordings in vitro tend to bias connectivity estimates. First, in slices some connections may be cut. Second, the recorded cells are typically located within a short distance of each other, but connections become sparser at longer distances and are thus more difficult to detect. Thus, the method tends to undersample connections at longer distances and mainly provides insight into local connectivity. Despite or perhaps even because of these limitations, the finding of local functional recurrent connectivity among L2S cells appears to be robust, and crucial for CAN models of grid cell firing. The relatively low connectivity percentage may suggest that excitatory input from other sources is needed for the generation of L2S grid cell firing.

#### 4.1.2. L2P → L2S.

Since pyramidal cells in all layers can also display grid cell firing, similar to L2S cells, it may be that the L2S cells inherit their grid firing from pyramidal cells. Indeed, L2S cells were found to receive remarkably strong monosynaptic glutamatergic projections from L2Ps: in 13.5% of recorded L2P-L2S pairs, L2P stimulation elicited an excitatory synaptic response in a L2S cell (52) (FIGURE 4, *A* and *D*). In vivo, optogenetic stimulation of L2P cells also appears to elicit responses in L2S cells: 50% of extracellularly recorded cells in L2 showed an excitatory response (65), and L2Ps make up at most 40% of principal cells in L2 (237). However, it is difficult to compare these in vivo results directly with the in vitro results above, since the optogenetic stimulation induces unphysiologically high synchrony among stimulated presynaptic cells, in contrast to the limited single-cell stimulation in vitro as discussed above. Furthermore, the relatively long latencies of excitatory responses measured in vivo (up to ∼10 ms) (65) suggest that these also included multisynaptic responses.

#### 4.1.3. L3P → L2S.

Besides this input from L2P cells, L2S cells were also shown to receive a relatively strong input from L3P cells (7%) (52) (FIGURE 4, *B* and *D*). These L3Ps are also connected to the deep layers (45, 59, 317). The high connectivity rate of L3Ps, combined with their slowly integrating EPSPs and high excitability, makes this cell type an ideal candidate for interlaminar communication in the MEC.

#### 4.1.4. L2P and L3P connectivity.

The finding that L2S cells receive input from pyramidal cells in L2 and L3 and L2S cells have only sparse recurrent connectivity among themselves, suggests the possibility that L2P or L3P cells are themselves recurrently connected, forming an excitatory CAN that can generate grid cell firing patterns, which then in turn are inherited by L2S grid cells. Glutamate uncaging suggests that L2Ps do indeed receive scattered inputs arising from L2 and L3, as well as from deeper layers of the MEC (45). However, local recurrent excitatory connections between L2Ps appear to be relatively rare (∼2%) (50, 52) (FIGURE 4*D*). Intermediate pyramidal cells as defined by Fuchs et al. (50) were more strongly connected with each other (5%) and with pure pyramidal cells (5–8%), suggesting an overall somewhat higher recurrent connectivity for L2Ps. Even higher connectivity rates were reported between L2Ps identified based on their morphology in an electron microscopic reconstruction study (22%, *n* = 54) (51). This may be explained by differences in the identification of L2Ps and the presence of weak synapses that cannot be easily detected in paired patch-clamp recordings, although the finding that pairs of cells were typically connected via multiple, clustered synapses (51) makes this possibility less likely. Despite these methodological considerations, overall the data suggest that functional, local recurrent excitatory connections between L2Ps are robust but relatively sparse. In turn, this suggests a relatively weak connectivity within L2P patches, which is surprising and very different from the strong recurrent connectivity (25–36%) within cortical barrels (313), which also arise from the clustering of cortical excitatory cells. L2Ps may be more specialized in connections over longer distances, either between different L2 patches (95) or between hemispheres (58).

Monosynaptic connections between L3Ps and L2Ps were also not found in either direction (0%) (52) (FIGURE 4*D*), suggesting that they do not form a recurrent excitatory network within superficial MEC. In contrast, recurrent connectivity among L3Ps is strong (6–9%) (44, 52). Thus, a CAN consisting of recurrently connected L3Ps could in theory generate grid cell firing, which is then inherited by L2S cells. How L2P cells are able to generate grid cell firing would remain unexplained in this scheme, however.

#### 4.1.5. L2S → L2P or L2S → L3P.

One alternative possibility is that the pyramidal cells in layers 2 and 3 are driven by L2S cells, thus forming a multisynaptic excitatory recurrent circuit (L2S → L2/3P → L2S) with CAN dynamics able to generate grid cell firing. This would require inputs from L2S cells onto L2/3 pyramidal cells, which were indeed reported in one study based on a small sample (8%, *n* = 24) (47). An optogenetic study reported that 14–19% of recorded pyramidal cells in layers 2 and 3 were depolarized in response to light stimulation of L2S cells in a Sim1-Cre mouse (see FIGURE 6*C*) (49). However, the unphysiological nature of optogenetic stimulation, together with the lack of detailed information on the timing of these responses (which were not the focus of the cited study), makes it difficult to compare the latter results with paired patch-clamp recordings, which consistently showed very sparse or absent connectivity (FIGURE 4*D*) when examining a larger sample of connections from L2Ss to L2Ps [0%, *n* = 126 (52); 0%, *n* = 77 (50)] or L3Ps [1%, *n* = 100 (52)]. Thus, a multisynaptic excitatory microcircuit linking L2P, L3P, and L2S cells appears unlikely.

On the other hand, the strong coherence among grid cells in a module (136, 222, 226), together with the fact that grid cells likely include not only L2S but also L2P and L3P cells (67, 127, 130, 249, 250), suggests that any CAN underlying grid firing in superficial MEC likely includes all three principal cell types. So how can L2P cells generate grid firing, if they do not receive any excitatory inputs (FIGURE 4*D*) from the other cells that are part of the assumed CAN? One explanation could be that grid firing in L2P cells is generated by mechanisms that are fundamentally different from those underlying CAN models: during a developmental phase, grid firing in L2Ps could be guided by mechanisms that combine spatially tuned input (for example from parasubiculum), synaptic plasticity, and cell-intrinsic dynamics (73–80). Such grid-tuned feedforward input to the MEC-hippocampal loop via L2P grid cells could supervise the development of recurrent connectivity underlying CAN dynamics (318, 319). In this way, spatially tuned activity of L2Ps would serve also to anchor the grids produced by CAN networks to physical space (48, 220, 320–323).

### 4.2. Inhibitory Connections in Superficial Layers

**FIGURE 5.**
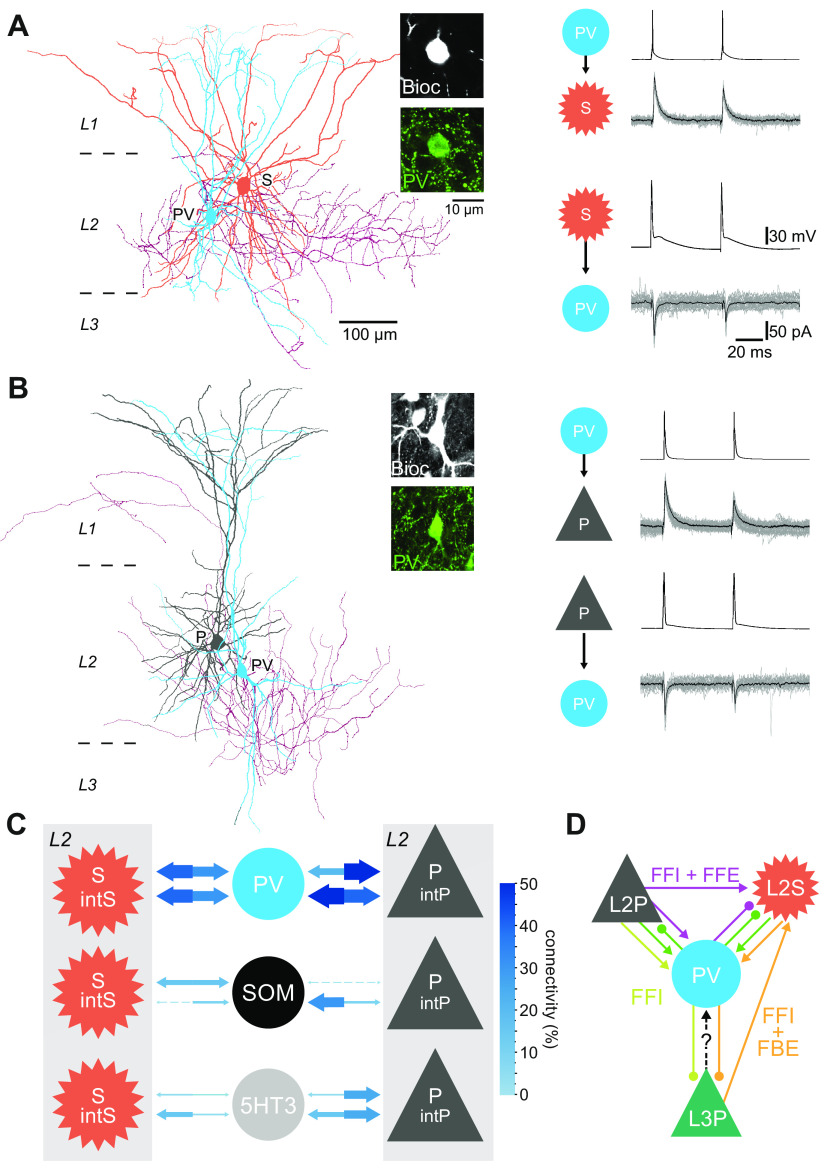
Superficial medial entorhinal cortex (MEC) inhibitory microcircuits. *A*: example paired recording from a layer 2 stellate (L2S) cell (red) and a fast-spiking basket cell (blue soma and dendrites, purple axon) expressing parvalbumin (*inset*, PV). Traces on *right* show that stimulation of the PV cell (*row 1*) induced a hyperpolarizing current in the L2S cell (*row 2*) and L2S cell stimulation (*row 3*) induced a depolarizing current in the PV cell (*row 4*). *B*: example paired recording from a layer 2 pyramidal (L2P) cell (gray) and a PV cell (same colors as above). Note again connectivity in both directions. Scales as in *A*. *C*: summary of inhibitory microcircuits for layer 2 principal cells. Blue arrows depict connectivity as % of postsynaptic cells showing a response to induced presynaptic spikes. Dashed lines represent 0% connectivity. PV cell connectivity with L2S and L2P cells is from Grosser et al. (292). All other data are from Fuchs et al. (50), who defined two additional types of layer 2 principal cells (intP, intermediate pyramidal cells; intS, intermediate stellate cells) and recorded from all 3 main classes of interneurons (SOM, somatostatin-expressing interneurons; 5HT3, serotonin receptor type 3a-expressing interneurons). Note that because of the difference in classification of layer 2 principal cells, it is not possible to directly compare the results from these 2 studies. *D*: motifs involving PV interneurons. Three motifs with feedforward inhibition (FFI) are apparent: *1*) from L2P to L2S, accompanied by feedforward excitation (FFI + FFE; purple); *2*) from L2P to layer 3 pyramidal cell (L3P), not accompanied by excitation in either direction (FFI; light green); *3*) from L2S to L3P, accompanied by feedback excitation (FFI + FBE; orange). In addition, 2 feedback inhibitory motifs can be discerned: 1 among L2S cells and 1 among L2P cells (both dark green); note that these feedback inhibitory motifs are both accompanied by sparser (1.7–6%) feedback excitation among the principal cells (FIGURE 4). Further motifs are likely to emerge, for instance if L3P cells provide input to PV interneurons, which has not been demonstrated directly (‘?’). A and B adapted from Grosser et al. (292) with permission from *eNeuro*.

CAN models depend on cells with similar tuning properties being interconnected, but these specific connections can in theory be excitatory, inhibitory, or a combination of both (71, 174, 282, 324–327). In paired recordings of L2S cells, Couey et al. (47) found that stimulation of L2S cells led to exclusively inhibitory responses in simultaneously recorded L2S cells, which were mediated by FS cells (likely PV+) but not low-threshold spiking (LTS) cells (likely SOM+); this finding led to a CAN model that could generate L2S grid cell firing depending solely on inhibitory connections, together with an external excitatory drive (see below). This connectivity has been repeatedly confirmed with paired recordings in vitro (FIGURE 5, *A* and *C*) (48, 50), with connectivity percentages of up to 78% for dorsal MEC (292).

L2P cells also show high connectivity with fast-spiking interneurons, although this appears to depend strongly on how L2P cells are defined: Grosser et al. (292) recently reported ∼20–50% connectivity depending on the direction (FIGURE 5*B*), whereas Fuchs et al. (50) reported 0% connectivity in both directions for their “pure” L2Ps but 37–48% with “intermediate” pyramidal cells (FIGURE 5*C*). In vivo, optogenetic stimulation of L2Ps inhibited firing of ∼75% of extracellularly recorded layer 2 principal cells, with relatively short delays (∼1–6 ms) (65). This very large proportion of cells with a reduction in firing rate, together with responses in many FS interneurons (65), suggests that in vivo strong FS cell-mediated inhibitory connectivity exists between L2P cells and other principal cells in layer 2, including mostly L2S cells.

Optogenetic stimulation of L2P cells also induced disynaptic inhibition in ∼80% of extracellularly recorded L3P cells (65). Strong interaction of L3P cells with PV+ interneurons, but not other interneuron subtypes (including SOM+, VIP+, and CR+ interneurons), was shown in an in vitro model of Up and Down states (328), suggesting that at least part of the inhibition reported by Zutshi et al. (65) could be mediated by PV+ cells. On the other hand, L3P cells were reported to receive less inhibition than principal cells in L2 (278), and the reduced intensity of PV+ neuropil in L3 [only roughly 50% of what is seen in L2 (46, 251)] suggests that this is likely due to weaker innervation by PV+ cells.

Although microcircuits with sufficient connectivity are certainly necessary for CAN models, more is required to generate spatial coding: CAN models of grid firing require directional and speed input able to move the activity around. This can lead to specific predictions about functional connections. For instance, a model based on inhibitory connectivity predicted that interneurons should display grid firing (48). So do cells with particular functional properties indeed show such signs of a CAN connectivity, rather than random connections? In vivo, this question was directly investigated in a landmark study applying extracellular tetrode recordings from optogenetically tagged PV+ interneurons and unidentified principal cells (61). A great advantage of this approach was that functional cell types such as grid and HD cells could be identified; a disadvantage is that connectivity could only be inferred based on correlations between spikes recorded from different cells (with a delay consistent with monosynaptic connections), which could also be due to a common (temporally offset) input (see also Ref. 329). Buetfering et al. (61) found that 12% of tagged PV+ cells appeared to receive input from grid cells (at least threefold more than from HD cells, unclassified spatial cells, conjunctive cells, or other PV+ cells), and grid cells appeared to target almost exclusively either tagged PV+ interneurons or untagged cells with a high firing rate, presumably also interneurons. These data are consistent with the sparse excitatory and strong inhibitory connectivity reported in vitro and appear to support an inhibitory CAN model. However, the fact that PV+ interneurons were not grid tuned, and did not receive inputs from similarly tuned grid cells (61), argues against a simple model in which similarly tuned grid cells within a CAN module selectively drive interneurons in a one-to-one fashion. A slightly more advanced model, in which interneurons are driven by excitatory uncorrelated spatial inputs, can in fact generate selective grid firing in excitatory neurons without grid firing in interneurons (330).

Regardless of the particular model that is implemented (329), the specific importance of PV+ interneurons for spatial coding was recently shown directly by chemogenetic silencing of PV+ interneurons in the MEC, which caused a reduction in grid and speed cell tuning (64). The fact that both of these cell types were affected may suggest that a reduction in speed tuning impaired the velocity-dependent translation of the activity bump in a CAN, leading to a random moving of the firing fields of grid cells.

In contrast, neither speed nor grid cells were affected by silencing of SOM+ interneurons (64). Instead, this manipulation altered the spatial selectivity of cells with discrete aperiodic firing fields. In vitro, only Fuchs et al. (50) has specifically quantified SOM+ interneuron connectivity in the MEC, finding that L2P and intermediate stellate cells receive zero input from SOM cells, whereas L2S and intermediate pyramidal cells do receive substantial input (12–14%) (FIGURE 5*C*). Overall the data suggest that for both L2P and L2S cells at least some subpopulation is getting direct inhibition from SOM+ interneurons, which could mediate the effects of silencing in the Miao et al. study (64).

The dissociation between the effects of silencing SOM+ and PV+ interneurons suggests they may be embedded in different functional networks, with PV+ being more associated with speed and grid cells and SOM+ more associated with nonperiodic spatially selective cells. The dissociation also suggests that connectivity between SOM+ and PV+ cells, which has been reported in other cortical areas, may not be significant in the MEC. Interestingly, neither HD nor border cells were affected by either manipulation, suggesting that they may be more driven by external inputs rather than being dependent on local microcircuits. Alternatively, they may be more dependent on 5HT3aR+ interneurons.

Regarding 5HT3aR interneurons in the MEC, very little is known in terms of physiology, anatomy, and function. Fuchs et al. (50) reported a high probability of inputs from 5HT3aR cells (∼25%) (FIGURE 5*C*) onto L2Ps, consistent with the prominent CCK basket cell terminals previously reported (58). L2S cells receive considerably less inhibition from this category of interneurons overall (50). All principal cell types in layer 2 appeared to provide modest output onto 5HT3aR+ cells (FIGURE 5*C*). Because 5HT3aR+ interneurons are a very heterogeneous group, it will be important to investigate the extent to which particular subtypes have different connectivity profiles. This point can also be applied more generally to all three main interneuron classes, which each contain several subtypes with likely very different roles within their local microcircuits.

### 4.3. Connections between Deep and Superficial Layers

**FIGURE 6.**
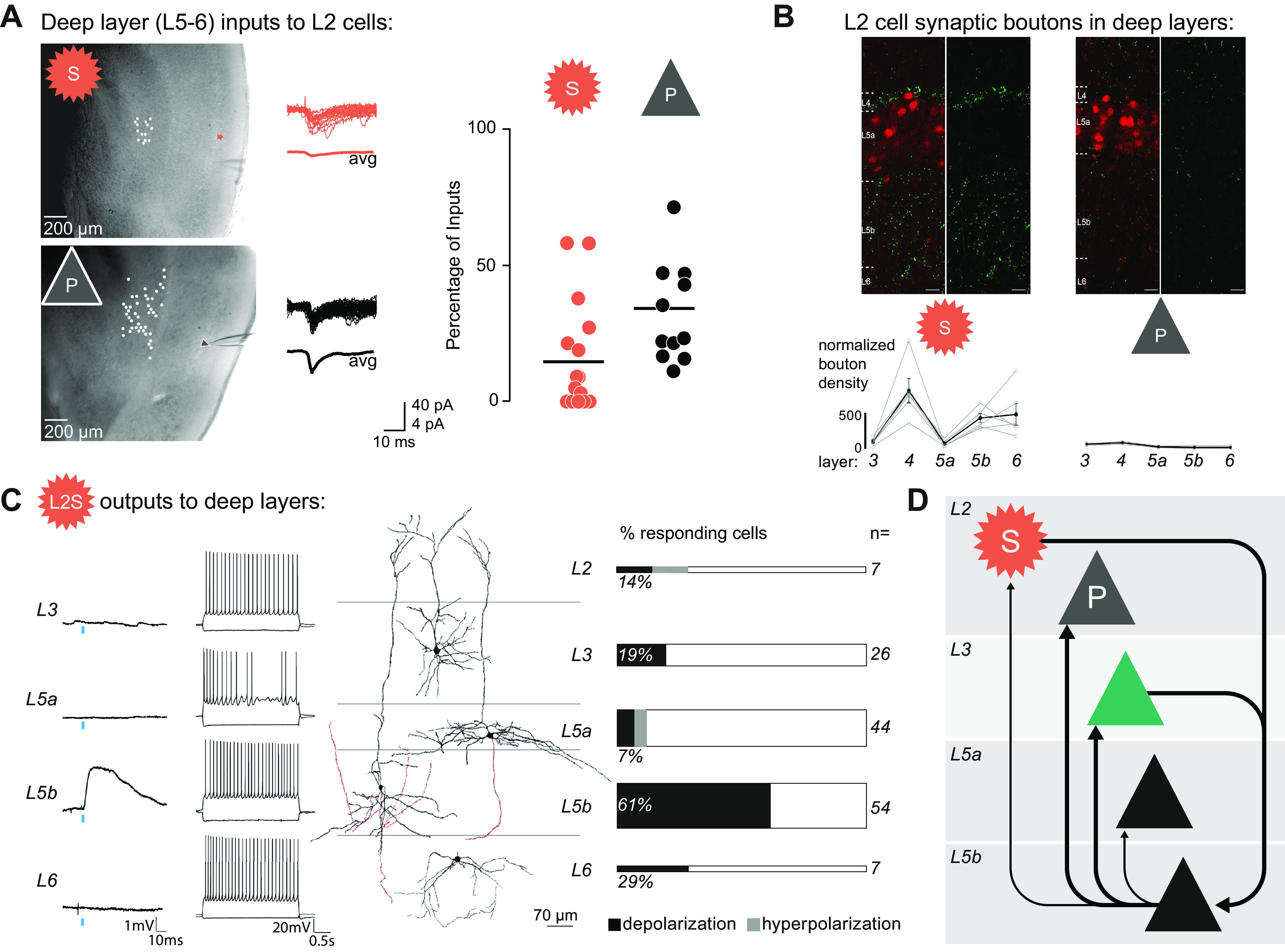
Superficial–deep microcircuits. *A*: example experiment showing glutamate uncaging in deep layers [*left*; dots indicate stimulation sites in medial entorhinal cortex (MEC) slices, symbols indicate intracellularly recorded cell in layer 2 (L2)] eliciting smaller average (avg) responses in a L2 stellate (L2S) cell (*top*, red) compared to a L2 pyramidal (L2P) cell (*bottom*, black). Population data (right) showing that L2P cells receive a greater percentage of input from deep cells than L2S cells. *B*: based on GFP-tagged synaptophysin, boutons from L2S cells (in Sim1-Cre mice) and L2P cells (in Wfs1-Cre mice) were detected, with a high density in most deep MEC layers for L2S cells (*left*) but virtually no synaptic boutons in these layers for L2P cells (*right*). *C*: example responses (*left*) from 4 cells recorded in different layers of the MEC to optogenetic stimulation (blue bar) of L2S cells (in Sim1-Cre mice). Note that only the L5b cell showed a membrane potential response. Morphologies and responses to current injection are also shown for all 4 example cells. Population data for all cells recorded in different layers are summarized on *right*, with L2 data coming from L2P cells only. Note that the height of the bars is proportional to the numbers of cells recorded. *D*: summary of the excitatory connectivity between deep layer and superficial layer principal cells. Note that we only indicate qualitative differences in the connections here and leave out connectivity within layers and amongst superficial layers (see FIGURE 4). *A* adapted from Beed et al. (45) with permission from *Neuron*. *B* and *C* adapted from Sürmeli et al. (49) with permission from *Neuron*.

So far we have discussed mostly superficial layer connectivity. However, there is also bidirectional communication between the superficial (L1–3) and deep (L5–6) layers in the MEC. In vivo, it was reported that deep layers have a large proportion of speed-modulated cells as well as conjunctive grid and head direction cells whereas the superficial layers have more distinct populations of head direction or grid cells (153). One hypothesis could be that the deep layers, which have been shown to have strong recurrent excitatory connections (44), might update the neuronal computations occurring in the superficial layers, based on the input they receive from the hippocampus (FIGURE 1*B*). Morphological reconstructions of L5 cells (331), anatomical tracer studies (59), as well as single-photon glutamate uncaging (45) (FIGURE 6*A*) indicate that deep layers provide input to the superficial layers in the MEC. In particular, L5b sends projections to layers 2 and 3 (59). L5b also receives synaptic inputs from L3P (317) and L2S neurons, whereas axon terminals from L2P neurons are very sparse in all deep layers (49) (FIGURE 6, *B* and *C*). Thus L5b cells appear to form excitatory loops with L3P and L2S cells (FIGURE 6*D*), which could in theory sustain an excitatory CAN network (although in fact L2P cells appear to receive a stronger input from deep layers than L2S cells) (45) (FIGURE 6, *A* and *D*). This loop can be expanded with hippocampal connections, as L2S and L3P cells provide the main output to the hippocampus (FIGURE 1*B*) and L5b was shown to receive the main input from the hippocampus (49). In contrast, L5a pyramidal cells receive very little input from L2 principal cells and project out of the MEC to other cortical areas like the retrosplenial cortex.

## 5. SUMMARY

Overall, the current data on local connectivity within the MEC, and particularly the superficial MEC, provide some constraints on possible anatomical instantiations of the CAN microcircuits thought to underlie grid cell firing. We summarize several main points:

- Grid cells in the superficial MEC likely include L2S, L2P, and L3S cells; therefore, for any functional CAN module at least a subset of these three cell types must be interconnected directly or indirectly.
- Monosynaptic recurrent excitatory connectivity within the L2S and L2P populations is present but sparse.
- Monosynaptic recurrent excitatory connectivity within the L3P and L5P populations is more abundant.
- Monosynaptic excitatory connectivity from pyramidal cells in both superficial layers onto L2S cells is abundant and unidirectional.
- Both L2S and L2P cells are strongly interconnected via PV interneurons and sparsely via 5HT3 interneurons. SOM input to L2P cells is sparse or absent.

## 6. OUTLOOK

Although many open questions remain, a start has been made in terms of describing the MEC microcircuits in vitro with multipatch approaches and in vivo with chemo- and optogenetics in combination with transgenic mouse lines. One major insight coming from recent studies on MEC microcircuitry is that there are not only feedforward but also recurrent excitatory and inhibitory connections within superficial layers 2 and 3 of MEC. It is tempting to speculate that recurrent connections enable CAN dynamics, which in turn contribute to spatial coding properties of cells in the MEC.

The extent to which synaptic connections underlying CAN dynamics in the MEC are excitatory or inhibitory, or some combination of both, remains an open question, but current data do suggest several possible polysynaptic motifs. Further anatomically precise models are needed to explore these possibilities. For instance, it is difficult to intuit whether 2% monosynaptic excitatory connectivity between L2S or L2P cells could be biologically significant for CAN function. Although 2% sounds extremely low, in hippocampal area CA3, classically considered to be a “highly recurrent” area, the connectivity rate among CA3b pyramidal cells was recently reported to be only ∼0.9% (146/15,930 tested pairs) and remarkably independent of the distance between the recorded cells (up to 400 µm) (332). The question of scale is crucial here: an overall low connectivity rate, such as 2% between L2Ps, can still be compatible with high local connectivity, which is all that is required for a CAN. Note that “local” here pertains to the connection matrix, not to the anatomical locations of neurons; even though paired patch-clamp recordings tend to be made from relatively nearby cells, one cannot know how local cells are in the connection matrix without recording very large numbers of cells from the same slice. In theory, the extent to which cells are locally connected should determine the size of the cell assembly representing the “bump size” of the CAN. Recent developments in technology for simultaneous recording of activity and connectivity from thousands of neurons at the same time, in behaving animals (333–339), will enable us to determine the minimal required connectivity of a functional CAN.

One way to address the issue of anatomically distributed cell assembly connectivity in vitro is to record a large number of pairs and identify higher-order motifs. In the Guzman et al. study (332), several higher-order motifs were identified, and it was shown that sparse connectivity specifically in combination with a disynaptic “chain” motif could generate pattern completion, a long-proposed function of CA3 recurrent networks (340–342). In general, multisynaptic motifs have been shown to occur in the cortex much more commonly than would be expected based on random connectivity (particularly among principal cells); such motifs have been posited to enhance memory storage capacity and could reflect associative synaptic plasticity underlying the formation of neuronal assemblies (314, 343–346). In the MEC, a high ratio of reciprocal connections has been shown directly between PV interneurons and L2S cells and L2P cells (47, 292), but the extent to which this exceeds chance levels (taking into account the extensive divergent and convergent properties of these cells) was not examined. In the neighboring presubiculum, which shares several properties with the MEC including the presence of grid cells (155), both reciprocal and “chain” motifs involving PV cells are statistically overrepresented (160).

Overall, the superficial MEC connectivity data reviewed here constrain possible motifs by ruling out, e.g., reciprocal connections between L2P cells and other principal cell types (FIGURE 4*D*). On the other hand, it also suggests several possible motifs, e.g., involving PV cells, that could be further investigated (FIGURE 5*D*). First, feedforward inhibition from L2Ps onto L2S cells appears to be accompanied by feedforward excitation of L2S cells, in a manner consistent with recent EM reconstructions (51). Second, feedforward inhibition from L2Ps onto L3P cells is not accompanied by feedforward or feedback excitation. Together, these motifs suggest that L2Ps may have a role in mediating feedforward input rather than participating in the recurrent circuits thought to form a local CAN in the superficial MEC. Third, feedforward inhibition from L2Ss onto L3Ps is accompanied by feedback excitation from L3Ps onto L2Ss. Fourth, feedforward inhibition from L2Ss onto L2Ps is also accompanied by feedback excitation. Finally, feedback inhibition is commonly seen among L2Ss and among L2Ps and is also likely to be present in L3Ps, although this has not been directly shown. The strong inhibitory connectivity among all principal cells suggests that the superficial MEC could form an inhibitory CAN that, if combined with a nonspecific excitatory drive, may be sufficient to generate grid cell firing (47). This is consistent with the finding that most PV cells contact both L2S and L2P cells (13). The excitatory drive may reach L2S cells via pyramidal cells in either layer (FIGURE 4*D*), which could mediate external inputs from the pre- and parasubiculum (FIGURE 1*B*) or from L5b (FIGURE 6*D*). L5b pyramidal cells in turn may be bound into the CAN via direct inputs from L2S (FIGURE 6, *C* and *D*), as well as relaying a drive from the hippocampus that has been shown to be important for grid cell function (148).

Beyond the feedback inhibition motif between L2S cells (47, 48), the role of specific network motifs in spatial coding has not been directly explored to our knowledge. The feedback inhibition motif at the network level could be instantiated at the neuron level as two different disynaptic motifs: a reciprocal motif between pairs of cells or a “chain” motif involving a principal cell, a PV cell, and another principal cell. It is likely that the ratio of such neuron motifs will have a large effect on for instance how effectively activity can spread within a network. As ever-higher numbers of cells can be simultaneously recorded in vitro, it will be interesting to see more detailed studies and models of MEC network topology, also including different types of interneurons. Possible motifs involving other interneuron types in the MEC remain difficult to identify since connectivity data on these cell types remain mostly limited to a single study, which did not include layer 3 and classified principal cells into four instead of two categories (FIGURE 5*C*) (50).

In general, classification of cell types remains an important challenge for the future, both for interneurons and principal cells (FIGURE 3). It has not been possible thus far to directly relate anatomically and functionally defined cell types, in large part because there is no straightforward link between anatomical (molecular expression profile, hodology, morphology, layer, brain region) and intrinsic physiological properties (50, 91, 232, 233, 289, 347–349). Adding further complexity, pyramidal cells in different MEC layers and even different parahippocampal areas can have similar functional properties (153, 155), and there can be substantial variability of function for any particular anatomically defined cell type both within and between animals [e.g., MEC L2 stellate cells (243)]. This is perhaps even more pronounced for different types of inhibitory interneurons, which can also have differential functional roles (61, 64, 199, 200).

One possible solution may be to start out with functionally defined cell types and determine their functional connectivity through precise manipulations in vivo. Precise optical stimulation methods with simultaneous imaging may enable experiments in the MEC that can indeed combine functional readouts based on calcium sensors with optogenetically driven tests of connectivity and function, as has been shown in other brain areas (333, 334, 337). These methods can overcome some of the shortcomings of optogenetics approaches applied in the MEC thus far, where typically large numbers of genetically identified cells were synchronously activated, in a highly nonphysiological manner, likely leading to overestimates of functional connectivity. However, the low temporal resolution of current calcium imaging limits their use in investigating monosynaptic connectivity compared to electrophysiological methods. By combining electrophysiological and imaging methods with optogenetics in behaving animals, it is in principle already possible (but challenging) to directly address the question of how grid cells and their underlying CAN neural circuits are connected to cells encoding speed, head direction, or landmark information. Speed and HD input must be input into the CAN in order for spatially stable grid patterns recorded from grid cells to arise: the stability of the grid firing can be seen as a representation or readout of a path integration process (71, 350). Although the path integration is in principle separate from the CAN dynamics, which are also present for instance in sleep (151, 152), the HD and speed inputs must be integrated in the same superficial MEC microcircuits underlying CAN dynamics. This information is likely passed on from cells in the pre- and parasubiculum, which encode speed, direction, and grid cell patterns reflecting already-integrated combinations of these two variables, onto cells in the superficial MEC (155, 158, 176, 181, 351). In addition, speed information is likely to come from the medial septum (206, 212), and other inputs from for instance the visual or retrosplenial cortex may also contribute. Besides needing to be able to continually keep track of speed and direction, CANs must also be periodically “anchored” to particular aspects of the environment, to prevent the accrual of random errors over time. Again the anatomical pathways mediating such inputs remain unclear: grid cell CANs may receive this input from local border or OV cells, or from extrinsic sources. Importantly, the output from the MEC into the hippocampus comes primarily from L2Ss and includes not only grid cells but many functional cell types (154, 250). Thus, it would be wrong to consider MEC merely as a circuit to produce grid cell output: other cell types recorded in the MEC are likely to also have roles beyond the generation of grid cell firing.

Approaches based on functional cell types in vivo are easily combined with genetic methods, enabling some insights into how anatomical cell types relate to functional cell types. Finding the anatomical substrates underlying function is particularly important for translational questions. For the next generation of drug development, a circuit-level understanding based on extracellular “units” needs to be complemented with concrete knowledge of which neurons should be targeted to effectively counteract pathological circuit function and what their molecular profiles are (e.g., specific receptors). MEC layer-specific and cell-type specific lines (347), possibly using intersectional approaches (352–354), could allow us to dissect the excitatory and inhibitory microcircuitry of the MEC in much finer detail. It is likely that more heterogeneity exists than the simple schemes we currently use, and perhaps higher-dimensional anatomical analyses are needed, as have been applied in visual cortex and other areas (286, 355–358). Combining such anatomical knowledge with in vivo functional readout and manipulation approaches as outlined above will surely provide great insights in the future. Alternatively, combining in vivo with in vitro approaches may enable even greater mechanistic insights into the anatomical underpinnings. In vivo, functional traits of cells can be determined with, e.g., patch-clamp or juxtacellular recordings enabling single-cell labeling or transfection (90, 359–361), head-fixed or head-mounted calcium or voltage imaging in combination with precise optogenetic stimulation (337) and virtual reality (362), or spatiotemporally controlled labeling or gene expression (363–367). Combining these approaches with post hoc identification of the functionally characterized cells enables further in vitro anatomical, electrophysiological, or even ultrastructural identification of the microcircuits in which they are embedded (51, 368–370). Complementary approaches recording from many cells at the same time are also likely to be required to decipher the circuit, via imaging approaches, high-density electrophysiological recordings, or a combination of both (371–377). Ultimately, such big-data approaches will enable much more complete models of the microcircuits underlying spatial coding. The insights from these models will enable artificial agents to navigate better, or at least help us understand the performance of artificial agents under some circumstances, as recently shown for a recurrent network trained to perform path integration, in which units developed gridlike properties as well as border, HD, and conjunctive tuning (378) (see also Ref. 379). This agent outperformed previous deep learning navigation systems, e.g., exploiting novel shortcuts in challenging environments. Perhaps more importantly, these models may shed light on general knowledge representation and memory processes and enable novel ways to improve circuit function for a wide range of pathologies (see *Clinical Highlights*) involving this fascinating brain area.

## GRANTS

This work was supported by the Stiftung Charité to P.B.; the DZNE, the Einstein Foundation, the German Research Council [Deutsche Forschungsgemeinschaft (DFG) project 184695641–SFB 958 to D.S., project 327654276–SFB 1315 to D.S. and R.K., and under Germany’s Excellence Strategy–Exc-2049-390688087 to D.S.]; the German Federal Ministry of Education and Research to R.K. (project 01GQ1705); and the Centre of Excellence scheme of the Research Council of Norway (Centre for Neural Computation, grant number 223262) and the Kavli Foundation to E.I.M.

## DISCLOSURES

No conflicts of interest, financial or otherwise, are declared by the authors.

## AUTHOR CONTRIBUTIONS

J.J.T. prepared figures; J.J.T., P.B., and D.S. drafted manuscript; J.J.T., P.B., M.B., R.K., E.I.M., and D.S. edited and revised manuscript; J.J.T., P.B., M.B., R.K., E.I.M., and D.S. approved final version of manuscript.

## Figures and Tables

**FIGURE A1.**
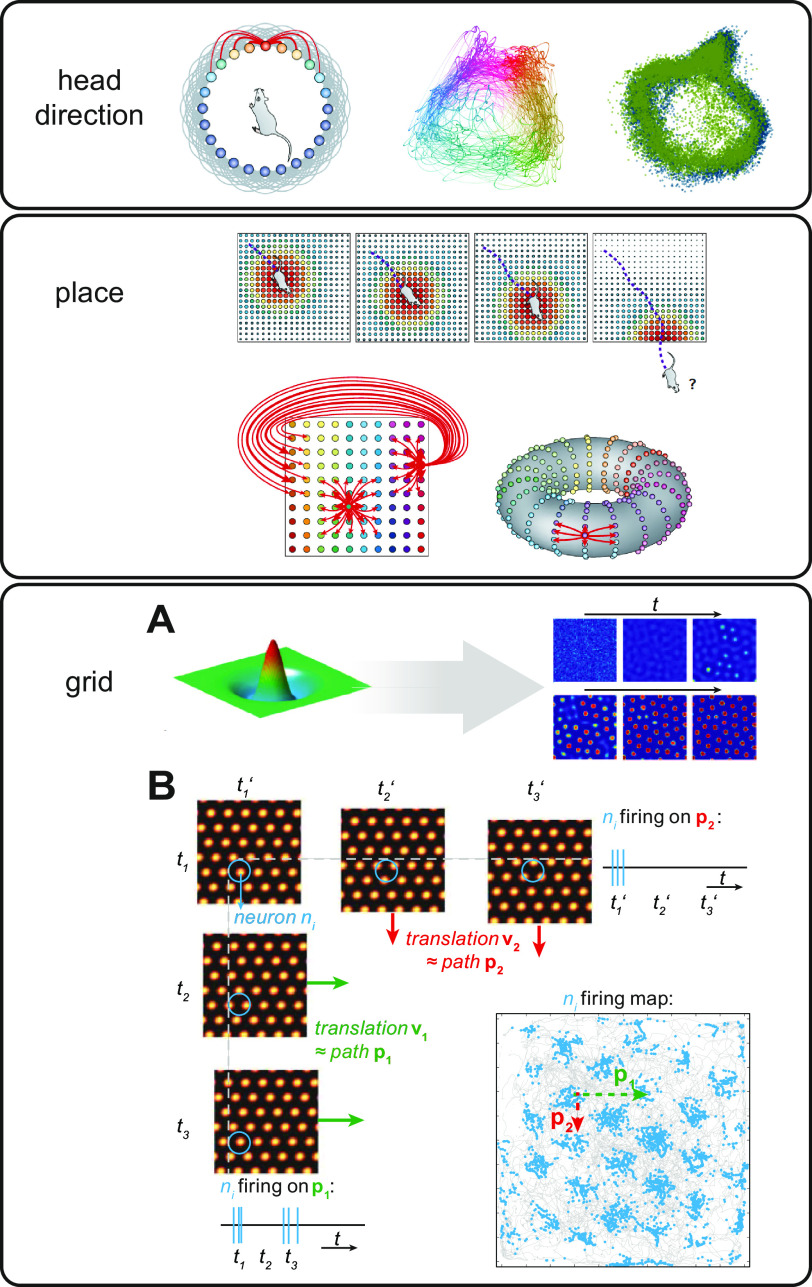
Continuous-attractor network (CAN) models of head direction (HD), place, and grid cell function. Head direction: A classic ring attractor network (*left*) in which neurons (circles) are connected (red, gray) such that only 1 bump of activity is active at a time (represented by warmer colors of circles), encoding the animal’s head direction. Note that additional mechanisms that are required to shift the activity appropriately when the head moves are not shown here. Activity of HD cells in the brain was recently shown (178–180) to indeed have a ring structure (*center*; colors represent different preferred head directions). This structure is very similar (*right*) during REM sleep (green) and awake (black) states (179). Place: A bump of activity in a 2-dimensional (2-D) sheet of hippocampal place cells can follow the animal’s position in space (*top*). To avoid discontinuity at the edge of the sheet, cells can be connected (*bottom left*; red arrows depict connections for 2 example cells) such that the CAN forms a torus (*bottom right*) (71). Grid: *A*: *a* specific connectivity profile (*top left*, in this case a combination of local excitation and surround inhibition) for neurons arranged on a 2-D sheet can lead to the emergence of a gridlike pattern of activity bumps (*top right*). Note that the connectivity profile shown is merely 1 example; different combinations of local and global excitation and inhibition can lead to a similar pattern. *B*: because the different states represented by the sheet are all connected on a 2-D manifold, the sheet can be easily shifted around from 1 state to another. Translation v follows the velocity of the animal’s locomotion, shown here for 2 example paths (p_1_, p_2_), each at 3 consecutive time points (*t*_1_, *t*_2_, *t*_3_ and *t*_1_′, *t*_2_′, *t*_3_′, respectively). The effect of these translations on neuronal firing is illustrated for 1 example neuron *n_i_*, both in the temporal (blue traces, *top right* and *bottom left*) and spatial (*bottom right*) domain. Note that in this example v_1_ is larger than v_2_, leading to a longer path for p_1_ than p_2_ (dashed arrows) in a shorter time (*t*_1_–*t*_3_ are closer together than *t*_1_′–*t*_3_′). In reality, the animal’s movement will contain a wide range of directions and speeds, but as long as the translations of the activity sheet closely mirror these, the pattern of firing for any single neuron will remain stable. Head direction left panel, all Place panels, and Grid panel *a* from McNaughton et al. (71) with permission from *Nature Reviews Neuroscience*. Head direction center panel from Rybakken et al. (180) with permission from *Neural Computation*. Head direction right panel from Chaudhuri et al. (179) with permission from *Nature Neuroscience*. Grid panel *B* inspired by Couey et al. (47) and Bonnevie et al. (148).

## APPENDIX: CONTINUOUS ATTRACTOR NETWORKS

A network of interconnected neurons can in principle reach a large number of states, often defined by “population vectors” of neuronal firing rates, which express the dynamic combination of activity in all cells of a recorded neuronal ensemble (380, 381). In many neural networks, the dynamics tend to spontaneously converge on only a limited number of stable states, known as attractors. If we assume that particular stable states in a memory network represent previously encoded activity states, then the shift of the network activity from some starting state to an attractor can be seen as memory recall (382). With an appropriate network architecture, several attractor states can be linked such that the transition between them has a very low threshold (0 in the extreme case of an infinite network), forming a “quasi-continuous” manifold of stable states, which can represent continuous variables (324)—now referred to as a CAN.

Theoretical insights like these gave rise to models of orientation tuning in neurons of the visual cortex (383) and memory of eye position in oculomotor circuits of the brain stem and cerebellum (384). Early on, it was also acknowledged that CANs provide powerful models of representation of self-location, both in one dimension, as expressed in head direction cells (172,173, 385), and in two dimensions, as in place cells (145, 223, 386, 387). Two-dimensional models for place cells (145, 223) were subsequently extended to account for grid cells (47, 71, 139, 282, 321; see 388 for a historical review). In these models, the CAN can be visualized as an arrangement of neurons with activity on a periodic manifold, where the cells are arranged such that the cells with most similar connectivity are closest together, whether this is on a circle, in one dimension, or on a torus, for a two-dimensional manifold (FIGURE A1). On these continua, the active cells at a given moment in time form a “bump” of activity. Because CANs contain a range of stable states, the bump can be easily moved around from one stable state to another, in accordance with external inputs to the CAN. Spontaneous noise-driven “drift” can occur in the absence of inputs that, over time, may lead to “errors” or mismatches with the position in space that they normally correspond to. Errors induced by drift in a CAN are expected to be coherent across all its elements, a prediction that has been confirmed in recordings from coactive neurons in the MEC (143, 147, 151,152).

In the case of spatial navigation, in order for the underlying neurons to remain linked to the same place and exhibit stable spatial coding, two aspects are needed. First, the bump of activity in the CAN must be moved in a manner that is consistent with the speed and direction of the animal’s movement; exactly how this is implemented in the brain remains an important topic of research. Furthermore, the CAN must be “anchored” to visual landmarks or other location-correlated sensory inputs in the external environment. This enables the animal to have different sets of active neurons for different environments. Location-correlated input can also serve to periodically correct the abovementioned drift and may serve to “teach” the network during development, such that the connectivity underlying CAN dynamics becomes linked to spatial coding properties (71, 318, 319).
